# Complement Receptor 3 Mediates HIV-1 Transcytosis across an Intact Cervical Epithelial Cell Barrier: New Insight into HIV Transmission in Women

**DOI:** 10.1128/mbio.02177-21

**Published:** 2022-01-11

**Authors:** Christopher J. Day, Rachael L. Hardison, Belinda L. Spillings, Jessica Poole, Joseph A. Jurcisek, Johnson Mak, Michael P. Jennings, Jennifer L. Edwards

**Affiliations:** a Institute for Glycomics, Griffith University, Southport, Queensland, Australia; b Center for Microbial Pathogenesis, The Abigail Wexner Research Institute at Nationwide Children’s Hospital, Columbus, Ohio, USA; c Department of Pediatrics, The Ohio State University, Columbus, Ohio, USA; Pasteur Institute

**Keywords:** HIV, complement receptor 3, cervical epithelial cells, transcytosis, carbamazepine, methyldopa, human immunodeficiency virus

## Abstract

Transmission of HIV across the mucosal surface of the female reproductive tract to engage subepithelial CD4-positive T cells is not fully understood. Cervical epithelial cells express complement receptor 3 (CR3) (integrin α_M_β_2_ or CD11b/CD18). In women, the bacterium Neisseria gonorrhoeae uses CR3 to invade the cervical epithelia to cause cervicitis. We hypothesized that HIV may also use CR3 to transcytose across the cervical epithelia. Here, we show that HIV-1 strains bound with high affinity to recombinant CR3 in biophysical assays. HIV-1 bound CR3 via the I-domain region of the CR3 alpha subunit, CD11b, and binding was dependent on HIV-1 N-linked glycans. Mannosylated glycans on the HIV surface were a high-affinity ligand for the I-domain. Man5 pentasaccharide, representative of HIV N-glycans, could compete with HIV-1 for CR3 binding. Using cellular assays, we show that HIV bound to CHO cells by a CR3-dependent mechanism. Antibodies to the CR3 I-domain or to the HIV-1 envelope glycoprotein blocked the binding of HIV-1 to primary human cervical epithelial (Pex) cells, indicating that CR3 was necessary and sufficient for HIV-1 adherence to Pex cells. Using Pex cells in a Transwell model system, we show that, following transcytosis across an intact Pex cell monolayer, HIV-1 is able to infect TZM-bl reporter cells. Targeting the HIV-CR3 interaction using antibodies, mannose-binding lectins, or CR3-binding small-molecule drugs blocked HIV transcytosis. These studies indicate that CR3/Pex may constitute an efficient pathway for HIV-1 transmission in women and also demonstrate strategies that may prevent transmission via this pathway.

## INTRODUCTION

Despite advancements in antiretroviral therapy and infection prevention strategies, the global burden of human immunodeficiency virus (HIV) infections remains high. In the majority of women (86%), the female reproductive tract (FRT) is the initial site of HIV exposure (1–3). Transmission across the FRT epithelium is a critical first step in the development of systemic disease, wherein HIV is able to access and to infect subepithelial immune cells, including T cells, which ultimately can lead to the development of AIDS (4). The mechanism(s) by which HIV breaches the epithelium of the FRT upon initial exposure is mainly thought to be passive paracellular migration at sites of physical mucosal microabrasions caused by intercourse (5). Notwithstanding, HIV transcytosis through vaginal (6), endometrial (7, 8), and oral and intestinal (9–11) epithelial cells (12) is also reported.

Binding to a host cell surface molecule (i.e., a receptor) can trigger pathogen uptake into the intracellular compartment of a target cell with, or without, subsequent transcytosis. Immune cell receptors (e.g., CD4, CCR5, and CXCR4) that interact with HIV are well studied (13); however, cell surface receptors that mediate HIV binding and/or its entry into epithelial cells, including cervical epithelial cells, are not fully elucidated. HIV gp120 (a component of the envelope protein) binds to the mannose receptor on vaginal epithelial cells (14), albeit this receptor, reportedly, is not expressed by the cervical epithelium (15). Heparan sulfate proteoglycans and galactosyl ceramide can act as binding partners for HIV-1 adherence to oral and tonsil epithelia, but blocking these receptors does not completely abolish HIV adherence or its internalization (11, 16, 17). This suggests a role for additional epithelial cell surface molecules in mediating HIV-1 entry.

Complement receptor 3 (CR3) is an integrin (α_M_β_2_ or CD11b/CD18) heterodimer that is highly expressed, and functional, on the ecto- and endocervix (18). Enhanced infection of complement-opsonized HIV via CR3 has been shown for dendritic cells, monocytes, and peripheral blood mononuclear cells (PBMCs) (19–25). However, a potential role for CR3 in the initial contact of HIV with the cervical epithelium has been overlooked (26–28).

The alpha subunit of CR3 (CD11b) contains an approximately 200-amino-acid insertion, called the I-domain (29). The I-domain modulates ligand binding (30–34), and the modular structure of CR3 allows differential cellular effects based on the recognition of specific ligands (35–37). Importantly, numerous human pathogens can subvert CR3 function(s) to establish infection and disease, including streptococci (38, 39), Listeria monocytogenes (40), Mycobacterium tuberculosis (41, 42), Bordetella pertussis (43, 44), Escherichia coli (45), Candida albicans (46), some flaviviruses (47, 48), Ross River virus (49), *Leishmania* (50, 51), and Toxoplasma gondii (52). As a specific example, the interaction occurring between an adhesin-linked carbohydrate on Neisseria gonorrhoeae and the CR3 I-domain (53, 54) triggers a signaling pathway in primary human cervical epithelial (Pex) cells that results in CR3 activation (55), the uptake of N. gonorrhoeae into Pex cells (18), the upregulation of CR3 on the Pex cell surface (56), and the intracellular replication of N. gonorrhoeae (57). Whether CR3 on cervical epithelial cells contributes to HIV transmission is not known. We, therefore, sought to investigate whether a direct interaction occurs between HIV-1 and CR3 and, if true, the potential role of this interaction in mediating transmission across the cervical epithelium.

## RESULTS

### HIV-1 binds CR3 with high affinity via an interaction between mannosylated HIV N-glycans and the I-domain region of the CR3 alpha subunit, CD11b.

Recent work shows that the I-domain region of the CR3 alpha subunit, CD11b, exhibits lectin activity for glycans terminating in galactose (54, 55). We performed surface plasmon resonance (SPR) studies to determine whether surface-exposed glycans on HIV could similarly mediate an interaction with the CR3 I-domain. Five strains of clade B HIV-1 were tested: 2 lab-adapted (NL4-3 and NL4-3 AD8) and 3 transmitted founder (T/F) strains (WITO, RHPA, and REJO). HIV virions were flowed over immobilized human recombinant I-domain (rI-domain) or recombinant CR3 (rCR3) protein. All five strains exhibited high-affinity binding to rI-domain and to rCR3 (Table 1; see Fig. S1 in the supplemental material), which demonstrates that the HIV-1–CR3 interaction was not strain dependent. The equilibrium dissociation constants (*K_d_*) for the lab-adapted and the T/F HIV-1 strains with rI-domain or with rCR3 were in the nanomolar range, indicating that a high-affinity interaction with CR3 occurred for each virus. The highest-affinity interaction was observed for strain WITO that had been propagated in primary human PBMCs and was increased more than 35-fold compared to its propagation in the tumorigenic cell line HEK293T. These data demonstrate that a direct interaction occurred between HIV-1 and CR3. Moreover, these data show that the I-domain of CD11b was sufficient to reproduce the HIV-1–CR3 interaction, indicating that the I-domain mediates HIV adherence to CR3.

**TABLE 1:** Surface plasmon resonance analysis of rI-domain and rCR3 for HIV-1 and ligands^*a*^

Strain or ligand and treatment	*K_d_* (nM) with:	
	Human rI-domain	Human rCR3
Strains		
NL4-3 AD8 (293T)	238.9 ± 85.3	336.89 ± 97.9
+PNGase F	No binding	No binding

NL4-3 (293T)	90.9 ± 54.4	191.8 ± 92.3
+PNGase F	No binding	No binding

RPHA (293T)	911.8 ± 190.1	249.4 ± 64.3
+PNGase F	No binding	No binding
		
REJO (293T)	265.0 ± 78.3	90.3 ± 26.6
+PNGase F	No binding	No binding

WITO (293T)	297.9 ± 33.5	241.6 ± 137.0
+PNGase F	No binding	No binding


WITO (PBMCs)	7.92 ± 6.15	10.61 ± 3.63
+PNGase F	No binding	No binding

Ligands		
Man5	193 ± 29.7	73.0 ± 1.31
Carbamazepine	2.12 ± 0.24	ND
Methyldopa	1.01 ± 0.09	ND

a Equilibrium dissociation constants (*K_d_*) were measured for the noted HIV-1 strains (treated [+PNGase] or untreated with PNGase F) and for small-molecule ligands (Man5, carbamazepine, α-methyldopa) flowed over immobilized rI-domain or rCR3. The mean *K_d_* ± SD from a minimum of two technical repeats on each of two independently produced flow cells is shown. In parentheses is shown the cell type used to produce the virus: either HEK293T immortalized kidney cells (293T) or peripheral blood mononuclear cells (PBMCs). Man5, Manα1-3(Manα1-6)Manα1-3(Manα1-6)Man. “No binding” indicates that no concentration-dependent interaction was observed at the ≤100 μM concentration of the flowed sample. ND, not determined. Representative sensorgrams of these interactions are shown in Fig. S1.

10.1128/mbio.02177-21.1 FIG S1 Representative sensorgrams of Table 1 interactions. (A) NL4-3 AD8 produced in HEK293T immortalized kidney cells interacting with the human rI-domain. (B) PNGase F-treated NL4-3 AD8 produced in HEK293T immortalized kidney cells interacting with the human rI-domain. (C) NL4-3 AD8 produced in HEK293T immortalized kidney cells interacting with the human rCR3. (D) PNGase F-treated NL4-3 AD8 produced in HEK293T immortalized kidney cells interacting with the human rCR3. (E) NL4-3 produced in HEK293T immortalized kidney cells interacting with the human rI-domain. (F) PNGase F-treated NL4-3 produced in HEK293T immortalized kidney cells interacting with the human rI-domain. (G) NL4-3 produced in HEK293T immortalized kidney cells interacting with the human rCR3. (H) PNGase F-treated NL4-3 produced in HEK293T immortalized kidney cells interacting with the human rCR3. (I) RPHA produced in HEK293T immortalized kidney cells interacting with the human rI-domain. (J) PNGase F-treated RPHA produced in HEK293T immortalized kidney cells interacting with the human rI-domain. (K) RPHA produced in HEK293T immortalized kidney cells interacting with the human rCR3. (L) PNGase F-treated RPHA produced in HEK293T immortalized kidney cells interacting with the human rCR3. (M) REJO produced in HEK293T immortalized kidney cells interacting with the human rI-domain. (N) PNGase F-treated REJO produced in HEK293T immortalized kidney cells interacting with the human rI-domain. (O) REJO produced in HEK293T immortalized kidney cells interacting with the human rCR3. (P) PNGase F-treated REJO produced in HEK293T immortalized kidney cells interacting with the human rCR3. (Q) WITO produced in HEK293T immortalized kidney cells interacting with the human rI-domain. (R) PNGase F-treated WITO produced in HEK293T immortalized kidney cells interacting with the human rI-domain. (S) WITO produced in HEK293T immortalized kidney cells interacting with the human rCR3. (T) PNGase F-treated WITO produced in HEK293T immortalized kidney cells interacting with the human rCR3. (U) WITO produced in peripheral blood mononuclear cells interacting with the human rI-domain. (V) PNGase F-treated WITO produced in peripheral blood mononuclear cells interacting with the human rI-domain. (W) WITO produced in peripheral blood mononuclear cells interacting with the human rCR3. (X) PNGase F-treated WITO produced in peripheral blood mononuclear cells interacting with the human rCR3. (Y) Man5 interacting with the rI-domain. (Z) Man5 interacting with rCR3. Download FIG S1, PDF file, 1.6 MB. Copyright © 2022 Day et al. 2022 Day et al. https://creativecommons.org/licenses/by/4.0/ This content is distributed under the terms of the Creative Commons Attribution 4.0 International license.

**FIG 1:**
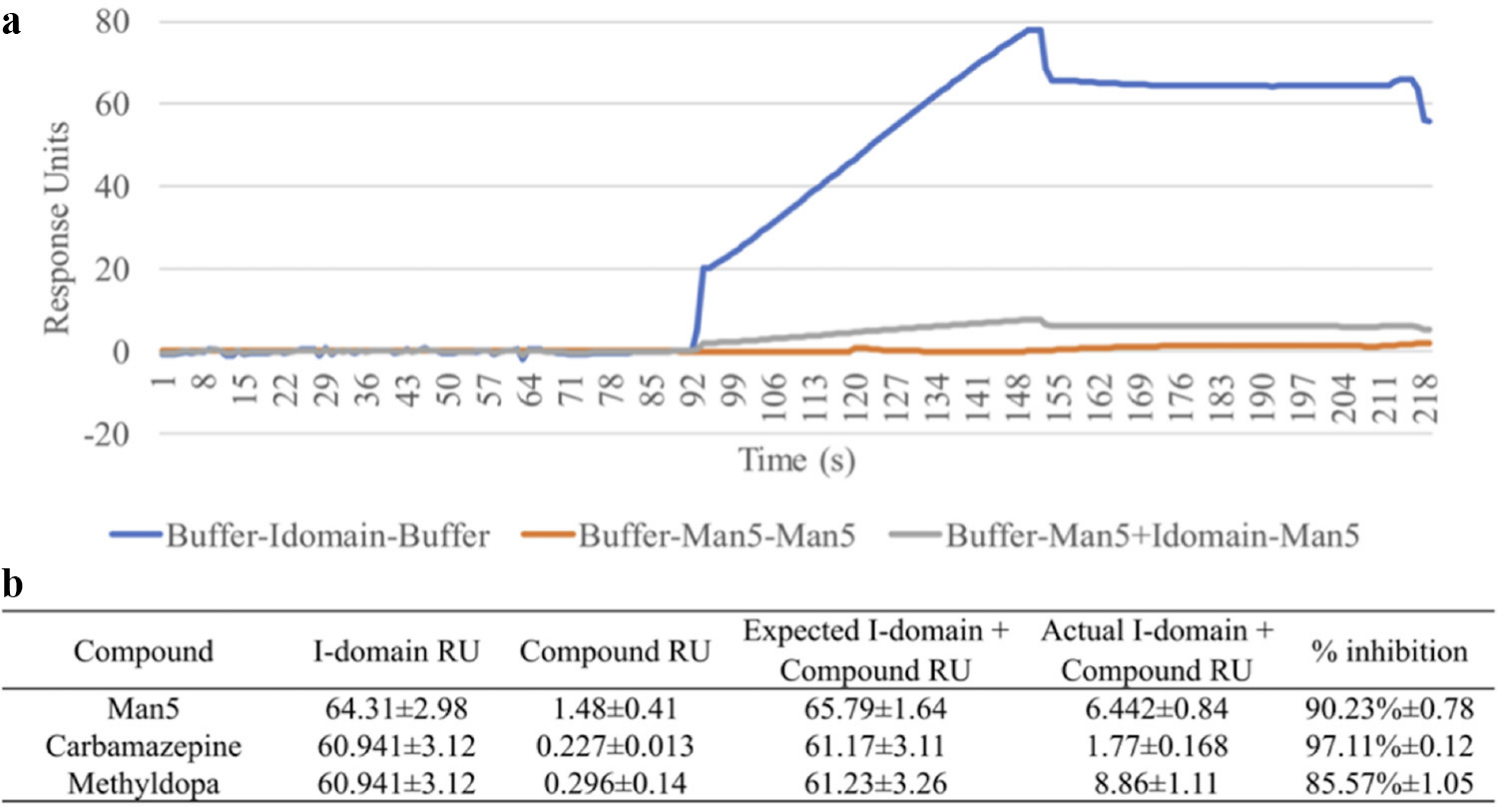
SPR analysis of the interaction between HIV and human CR3 I-domain in competition with the mannose pentasaccharide Man5. (a) Competition curves between Man5 and human rCR3 I-domain at 1 μM concentration. I-domain, Man5, or I-domain preincubated with Man5 was injected over immobilized HIV-1 WITO generated in primary human PBMCs. (b) Table of average response units (RU) from the interaction between immobilized HIV-1 WITO and flowed analytes (I-domain, Man5, carbamazepine, or α-methyldopa) alone, the expected additive binding response of competing analytes (I-domain plus either Man5, carbamazepine or α-methyldopa), the actual joint binding response of competing analytes (I-domain plus either Man5, carbamazepine, or α-methyldopa), and the percentage of binding inhibition of the I-domain to HIV in the competing interaction. Additional binding sensorgrams for these competition assays are provided in Fig. S2.

The I-domain of human CD11b has lectin activity in that it exhibits high-affinity binding for glycans terminating in galactose (54). The HIV envelope protein (Env) (gp120/gp41) is known to be heavily glycosylated with high mannose, which forms a “glycan shield” (58) at the virus surface. In particular, a highly conserved region of densely packed, predominantly high-mannose-type, glycans are present within the glycan shield and is termed the “mannose patch” (59, 60). We, therefore, hypothesized that the high-affinity interaction observed between HIV-1 and CR3 may be driven by HIV high-mannose N-glycans binding to the I-domain of CD11b. To test this hypothesis, we first measured the affinity of rI-domain or rCR3 for Man5, a mannose pentasaccharide that has a structure typical of those glycans found within the mannose patch. As a second approach, we removed (mannose) N-linked glycans from the HIV surface using protein *N*-glycosidase (PNGase F) before SPR analysis. Both rI-domain and rCR3 bound Man5 with high affinity (Table 1; Fig. S1), and N-glycan removal by PNGase resulted in the loss of a concentration-dependent interaction of HIV-1 with both the rI-domain and rCR3 (Table 1). We then conducted SPR studies with the Man5 pentasaccharide in competition (at 1 μM equal molar concentrations) with HIV-1 for binding to rI-domain. These studies demonstrated that Man5 could successfully compete with HIV-1 WITO propagated in human PBMCs for binding to rI-domain (Fig. 1; see Fig. S2 in the supplemental material). An ∼90% reduction in HIV binding to rI-domain occurred in the presence of Man5. Similar studies were performed in the presence and absence of the CR3 I-domain-blocking drugs carbamazepine and α-methyldopa and demonstrated blocking of HIV-1 binding to rI-domain (Fig. 1B; Fig. S2). Taken together, these data indicate that mannose N-linked glycans on HIV-1 were necessary and sufficient for the binding of HIV to the I-domain of CR3.

10.1128/mbio.02177-21.2 FIG S2 Representative competition curves in support of Fig. 1. Competition between 1 μM Man5 (A), carbamazepine (B), or α-methyldopa (C) and recombinant human CR3 I-domain at 1 μM concentration with immobilized HIV-1 WITO generated in primary human PBMCs. Download FIG S2, PDF file, 0.10 MB. Copyright © 2022 Day et al. 2022 Day et al. https://creativecommons.org/licenses/by/4.0/ This content is distributed under the terms of the Creative Commons Attribution 4.0 International license.

### HIV-1 adheres to host cells by a CR3-dependent mechanism.

**FIG 2:**
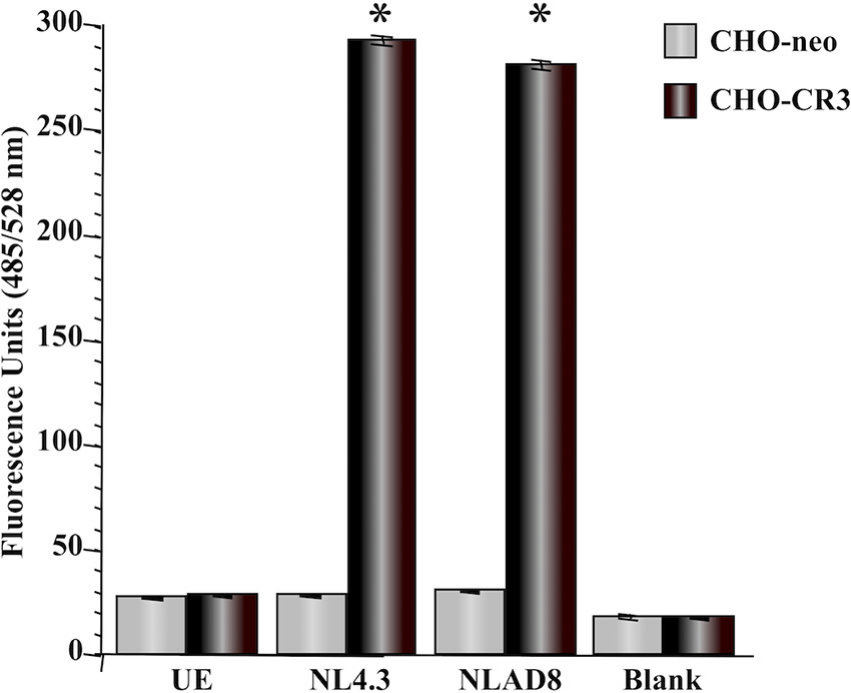
HIV-1 adheres to CHO cells in a CR3-dependent manner. Fluorometric adherence assays were performed as described in the text using fixed, GFP-tagged HIV-1 strains NL4.3 and NL4-3 AD8 (NLAD8) to challenge CHO cells that do (CHO-CR3) or do not (CHO-neo) express human CR3. Fluorescence (*y* axis), indicative of adherence, was recorded following a 2-h exposure and extensive washing of the host cell monolayers. HIV adhered to CHO-CR3 cells but not to CHO-neo cells, which demonstrates a CR3-dependent mechanism of adherence. Data are presented as the mean and variance of 3 assays performed in triplicate. UE, cells not exposed to HIV. *, *P* ≤ 0.0001 versus unexposed cells, blank wells (devoid of CHO cells, inoculated with HIV-1), and assays performed using CHO-neo cells (all comparisons).

To determine whether HIV binds to CR3 in a biological system, we performed fluorometric adherence assays. CR3-expressing (CHO-CR3) and -nonexpressing (CHO-neo) Chinese hamster ovary (CHO) cells were seeded onto microtiter plates, and adherence of fixed, green fluorescent protein (GFP)-tagged HIV-1 strains NL4-3 and NL4-3 AD8 was quantitated fluorometrically. Inoculation of CHO-CR3 cells resulted in a significant (*P* ≤ 0.0001) increase in relative fluorescence, indicative of HIV adherence. A greater than 9-fold increase in fluorescence was recorded for CHO-CR3 cells exposed to HIV compared to unexposed cells or to CHO-neo cells (Fig. 2). Incubation of CHO-neo cells with HIV resulted in a level of fluorescence that was not significantly (*P* ≥ 0.12) different from that recorded for uninoculated cells (Fig. 2). These results demonstrate that HIV-1 adherence to CHO cells required the expression of CR3.

**FIG 3:**
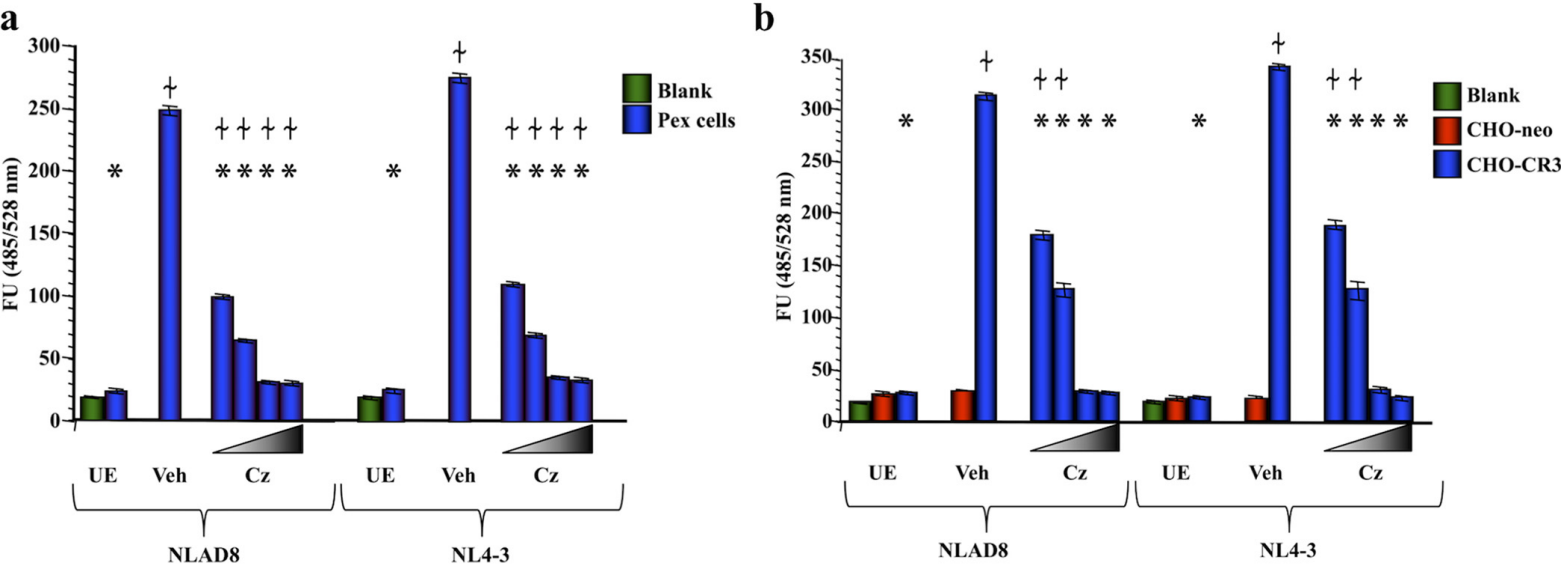
HIV-1 binds to Pex and CHO cells via the CR3 I-domain. Relative adherence of the lab-derived HIV-1 strains NL4-3 AD8 (NLAD8) and NL4-3 to Pex cells (a) and CHO cells (b) was determined using a fluorometric adherence assay. Fluorescence units (FU), indicative of HIV adherence, were recorded at 2 h postexposure in the presence/absence of 100 pM, 10 nM, 1 μM, or 100 μM the CR3 I-domain-binding drug carbamazepine (Cz). The data shown represent the mean and variance of 3 assays performed in triplicate. Blank, wells devoid of host cells but inoculated with HIV-1; UE, cells not exposed to HIV-1; Veh, DMSO vehicle control. *, *P* ≤ 0.0001 versus vehicle control; ≁, *P* ≤ 0.01 versus cells not exposed to HIV-1.

To confirm that HIV adherence was dependent upon CR3, as well as to determine the potential biological significance of this interaction as it relates to HIV infection in females, we performed competitive fluorometric adherence assays in which HIV adherence to Pex and to CHO-CR3 cells was comparatively evaluated in the presence and absence of the CR3 I-domain-blocking drug carbamazepine (Fig. 3) (54). In the absence of carbamazepine, HIV-1 strains NL4-3 and NL4-3 AD8 adhered to Pex cells at a similar level (Fig. 3a). In the presence of carbamazepine, a significant (*P* ≤ 0.0001 for all concentrations tested), dose-dependent decrease in adherence was observed. Adherence of both HIV strains was impaired by greater than 87% with ≥1 μM carbamazepine, consistent with adherence to the CR3 I-domain serving as a key mechanism by which HIV-1 adheres to human cervical epithelial cells. Carbamazepine also significantly (*P* ≤ 0.0001) blocked HIV adherence to CHO-CR3 cells in a dose-dependent manner, whereas HIV adherence to CHO-neo control cells was not significantly (*P* ≥ 0.6682) different than that recorded for uninoculated cells (Fig. 3b).

**FIG 4:**
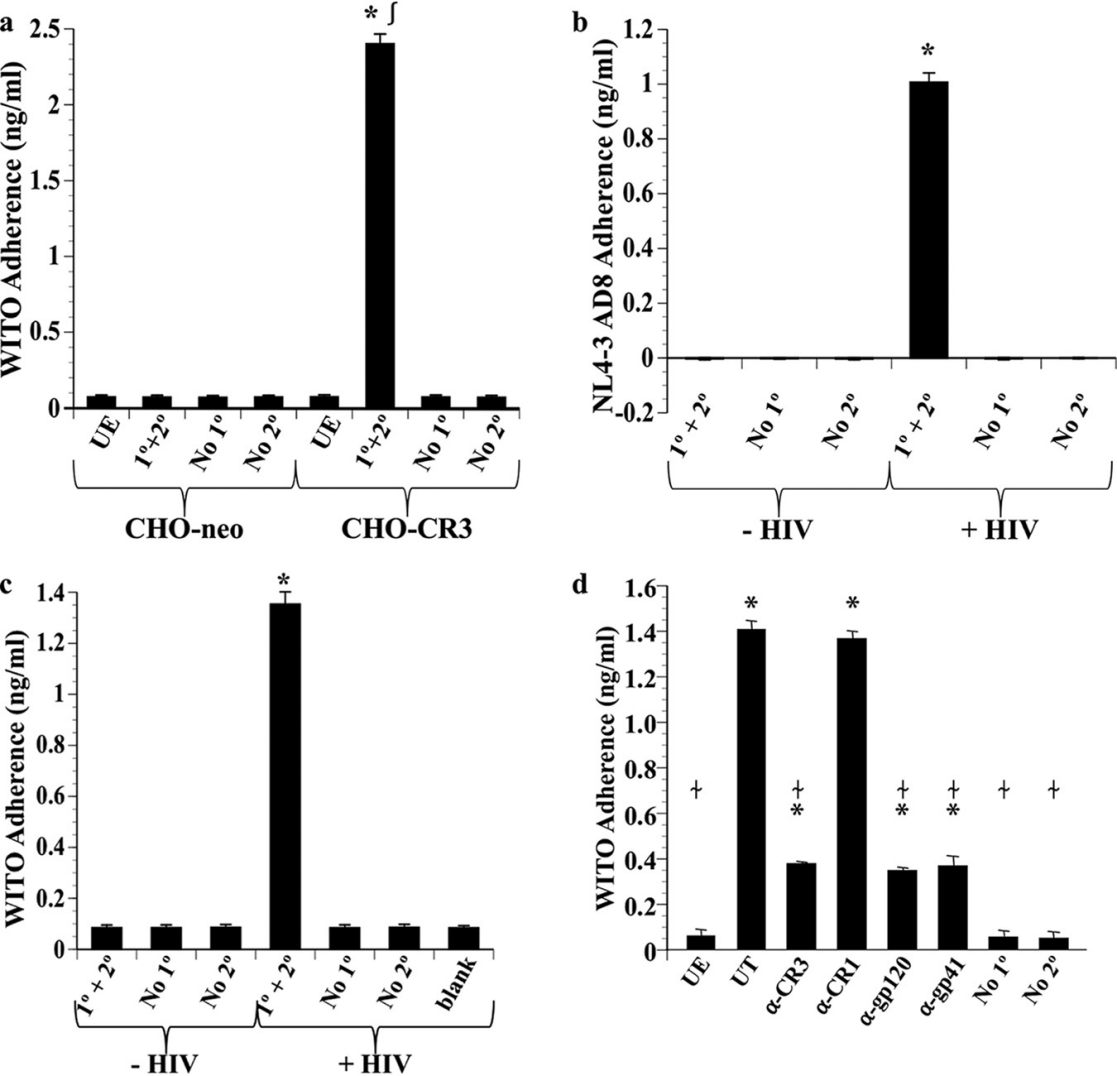
CR3-dependent adherence of HIV-1 to Pex and CHO cells. Adherence of HIV-1 to CHO cells (a) and Pex cells (b to d) was determined following immunolabeling and quantification against a standard curve (see Materials and Methods). (a) The T/F strain WITO bound to CHO cells in a CR3-dependent manner. Both lab-derived (NL4-3 AD8 [NLAD8]) (b) and T/F (WITO) (c) strains of HIV-1 bound to Pex cells. (d) Adherence of strain WITO to Pex cells was significantly decreased in the presence of monoclonal antibodies with the potential to block an HIV-CR3 interaction. However, the presence of a monoclonal antibody to complement receptor 1 (CR1) had no significant effect on HIV adherence. The mouse (blocking) antibodies used (d) comprised LM2/1 to CR3, E-11 to CR1, A00019.01 to HIV gp120, or A00020.01 to HIV gp41. The data shown represent the mean and variance of 3 assays performed in triplicate. UE, cells not exposed to HIV-1 (a and d); UT, untreated—no blocking antibody was included in the assay (d); 1° + 2°, immunolabeling included incubations with both the primary and secondary antibodies; No 1°, the primary antibody was omitted from the immunolabeling procedure; No 2°, the secondary antibody was omitted from the immunolabeling procedure. *, *P* ≤ 0.0001 versus unexposed (−HIV) cells; ∫, *P* ≤ 0.0001 versus CHO-neo cells (a); ≁, *P* ≤ 0.01 versus untreated cells (d).

**FIG 5:**
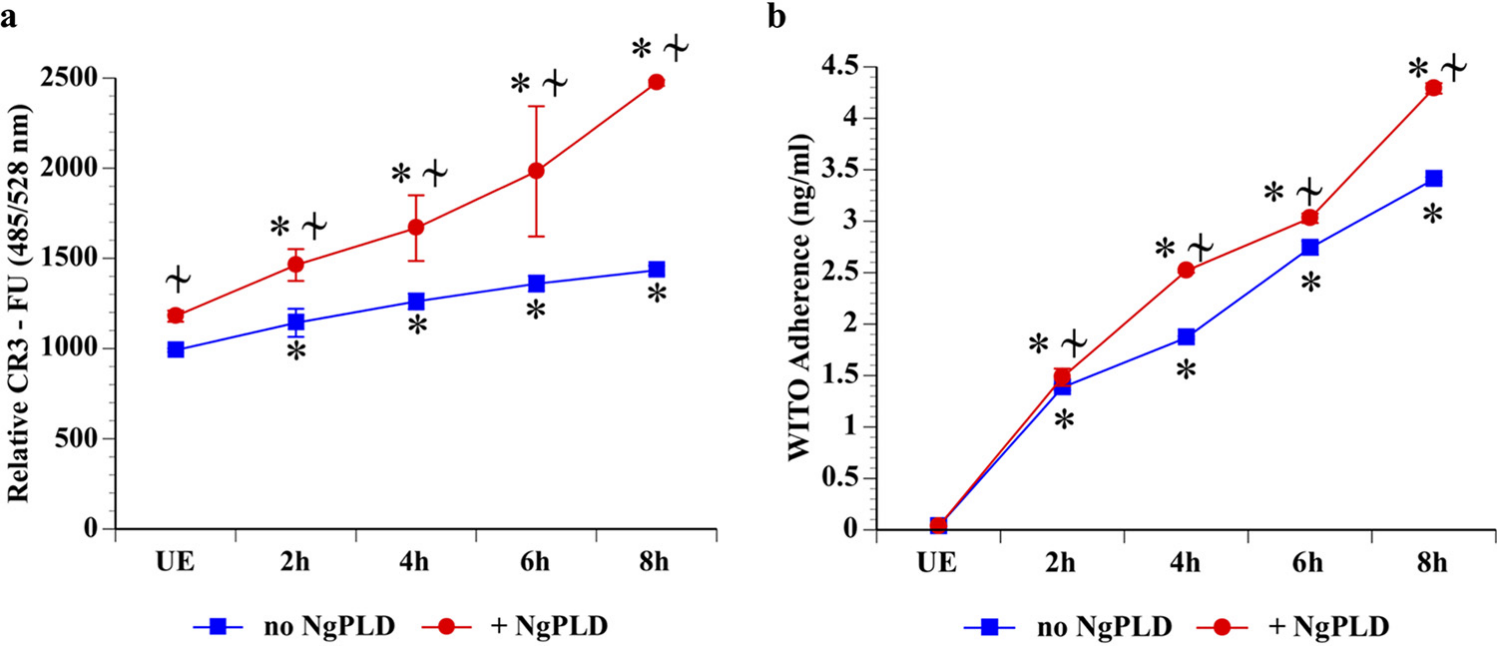
HIV-1 exposure increases CR3 surface expression by Pex cells. (a) The relative expression of CR3 on the Pex cell surface was measured over the course of an 8-h exposure to the T/F HIV-1 strain WITO. CR3 expression was recorded as fluorescence units (FU) and was correlated with HIV adherence (b). Both CR3 surface expression (a) and HIV adherence to Pex cells (b) were augmented in the presence of N. gonorrhoeae PLD (NgPLD). The data shown are the mean and the variance from 3 assays performed in triplicate. UE, cells not exposed to HIV. *, *P* ≤ 0.0001 versus cells not exposed to HIV; ≁, *P* ≤ 0.0196 versus the absence of NgPLD.

We modified and repeated our fluorometric adherence assays to allow quantification of HIV adherence. Toward this end, fluorescence was recorded following HIV immunolabeling, and HIV was quantified using a standard curve, as described below. Cells were exposed to 10 ng/mL (p24 capsid HIV equivalent) the lab-derived HIV-1 strain NL4-3 AD8 or the T/F strain WITO for 2 h. In all assays, only background levels of fluorescence were recorded for CHO-neo cells, for CHO or Pex cells not exposed to HIV, or when the primary or secondary antibodies were omitted from the immunolabeling procedure (Fig. 4 and 5; see Fig. S3 in the supplemental material).

10.1128/mbio.02177-21.3 FIG S3 HIV-1 exposure results in the increased expression of CR3 on the Pex cell surface. Pex cells were exposed to HIV-1 strain WITO for 2 h, after which relative CR3 expression on the Pex cell surface was determined by immunolabeling CR3 and recording fluorescence units (FU). Pex cells not exposed to HIV (− HIV) expressed CR3, which was significantly increased following HIV exposure (+ HIV). The data shown represent the mean and variance of 3 assays performed in triplicate. UE, cells not exposed to HIV-1 strain WITO; No 1°, the primary antibody was omitted from the immunolabeling procedure; No 2°, the secondary antibody was omitted from the immunolabeling procedure. *, *P* ≤ 0.0001 versus unexposed (− HIV) cells. Download FIG S3, PDF file, 0.08 MB. Copyright © 2022 Day et al. 2022 Day et al. https://creativecommons.org/licenses/by/4.0/ This content is distributed under the terms of the Creative Commons Attribution 4.0 International license.

Exposure of CHO-CR3 cells to WITO resulted in approximately 24% (2.40 ± 0.06 ng/mL) viral adherence compared to the inoculum (Fig. 4a). A lower level of adherence was observed for Pex cells inoculated with HIV strain NL4-3 AD8 (∼10%; 1.01 ± 0.03 ng/mL) (Fig. 4b) or WITO (∼13%; 1.35 ± 0.05 ng/mL) (Fig. 4c), which was not surprising, given CHO-CR3 cells are designed to overexpress CR3. To verify that HIV adherence to Pex cells was dependent upon CR3, we pretreated Pex cells or strain WITO with antibodies with the potential to block a CR3-HIV interaction (Fig. 4d). Pretreatment of Pex cells with an antibody to the CR3 I-domain resulted in significantly reduced (∼73%; *P* ≤ 0.0001) adherence compared to untreated, HIV-exposed Pex cells. Similar results were obtained when we pretreated WITO with anti-HIV gp120 or -HIV gp41 antibodies. In contrast, pretreatment of Pex cells with an antibody to complement receptor 1 (i.e., CD35) had no significant (*P* ≥ 0.6682) effect on the ability of HIV to bind to Pex cells.

Taken together, our results demonstrate that HIV-1 bound to Pex cells via an interaction between HIV Env and the CR3 I-domain. These data reveal a potential role for CR3 in receptor-mediated HIV-1 transmission following initial HIV-1 exposure in women.

### HIV-1 exposure augments CR3 expression on host cells.

N. gonorrhoeae cells secret a phospholipase D (NgPLD) that upregulates the surface expression of CR3 on Pex cells (56). Given that N. gonorrhoeae infection is a risk factor for HIV-1 infection, and vice versa, this led us to question what effect HIV exposure might have on CR3 surface expression by cervical cells. To address this question, relative CR3 surface expression on HIV-exposed and unexposed Pex cells was measured fluorometrically (55). CR3 surface expression was increased approximately 32% following a 2-h exposure of Pex cells to the T/F strain WITO, compared to unexposed cells (Fig. S3). We then measured (in parallel) CR3 expression by, as well as HIV adherence to, Pex cells in the presence and absence of NgPLD. An increase in both CR3 expression (Fig. 5a) as well as WITO adherence (Fig. 5b) was observed over the course of an 8-h exposure of Pex cells to HIV-1. HIV adherence was strongly positively correlated with CR3 expression (*r_s_* = 1; *P* [2-tailed] = 0), with or without the presence of NgPLD, and both HIV adherence and CR3 expression were significantly (*P* ≤ 0.0196) increased by the presence of NgPLD. Thus, these data suggest that an increase in CR3 expression on the cervical surface could potentially aid HIV-1 infection and also that this effect is likely augmented during HIV-1–N. gonorrhoeae coinfection.

### HIV-1 transcytosis across the cervical epithelium is mediated by CR3.

**FIG 6:**
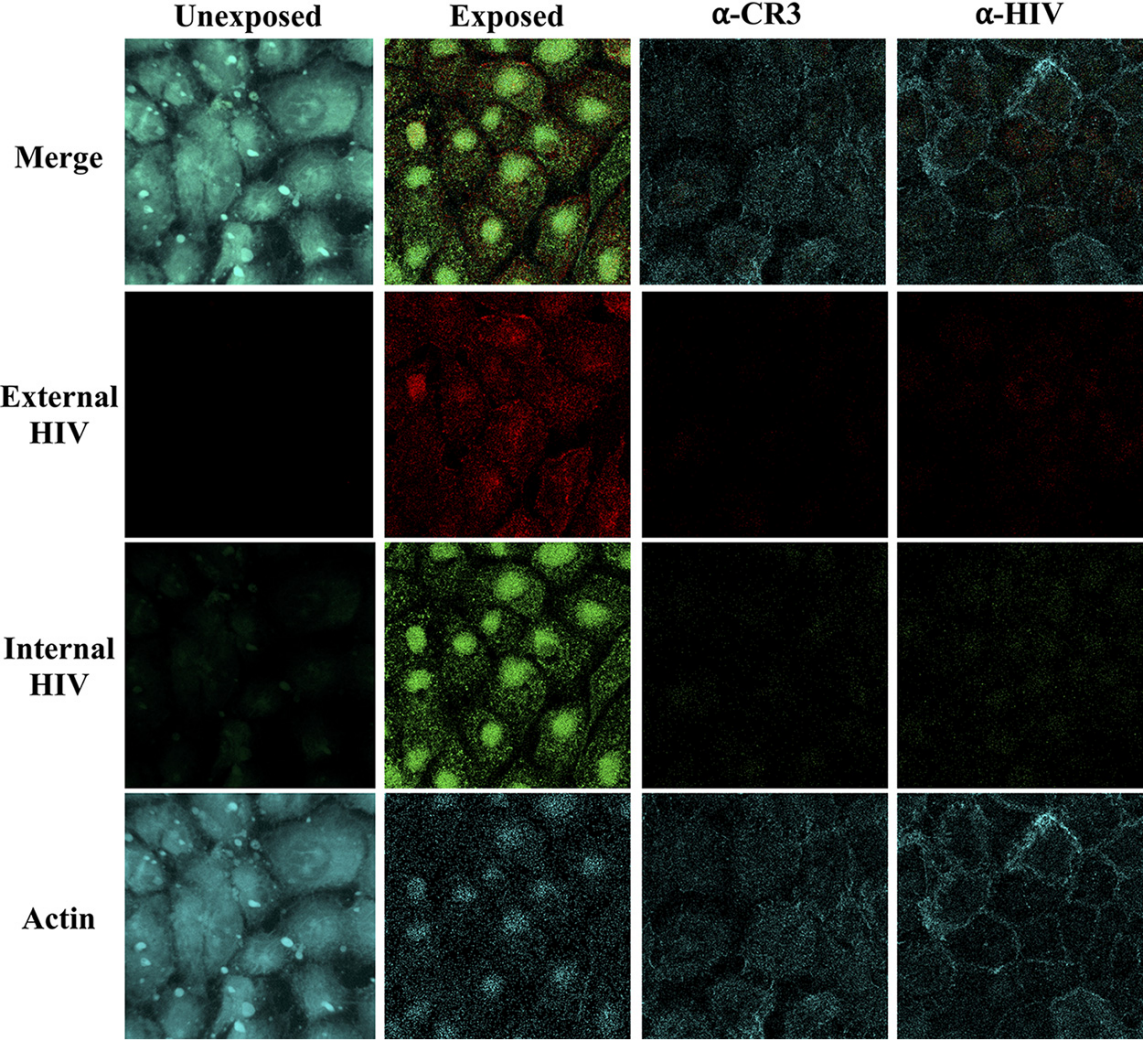
HIV-1 internalization into Pex cells is mediated by a CR3-Env interaction. Confocal microscopy was used to qualify HIV-1 strain WITO internalization into Pex cells following a 4-h exposure. External virus is visible as red fluorescence, whereas internal virus is visible as green fluorescence. Alexa Fluor 405-phalloidin (turquoise) was used to stain Pex cells. Negligible to low Pex cell autofluorescence was observed in the green channel (i.e., row labeled “internal HIV”) for all coverslips examined. External virus and internal virus were readily detected in Pex cells exposed to HIV-1, which was not observed in nonexposed Pex cells. The association of HIV-1 with Pex cells was dramatically reduced when either host cells or virus was pretreated with anti-CR3 (α-CR3) or anti-HIV (α-HIV) antibodies, respectively, to block the CR3 I-domain–HIV-1 interaction, as indicated by substantively reduced fluorescence in both the red (i.e., row labeled “external HIV”) and green channels. Images corresponding to assay controls are provided in Fig. S5 and are described in the text. Magnification, 63× with oil immersion.

Following adherence, numerous human pathogens also use CR3 as a mechanism to enter host cells (18, 35–38, 40, 42, 44, 51–53, 61). Therefore, we next asked whether CR3 could mediate HIV entry into and transcytosis across Pex cells. Toward this end, we first exposed Pex cells to HIV-1 and then performed a double-labeling fluorometric assay to distinguish extracellular HIV from intracellular HIV. The relative percentage of internal versus external HIV was measured at select intervals over a 24-h exposure. These data revealed that HIV strain WITO was rapidly and efficiently taken up by Pex cells, with approximately 40% of HIV being internalized by 2 h postexposure (see Fig. S4 in the supplemental material). Confocal microscopy confirmed the intracellular location of HIV-1 within Pex cells. Virus was visible on the extracellular surface and throughout the Pex cell cytoplasm, with a particular perinuclear localization (Fig. 6). Only minimal fluorescence was observed when Pex cells or virus was pretreated with antibodies with the potential to block the CR3 I-domain–HIV interaction (Fig. 6), providing further support for the idea that CR3 mediates HIV internalization into Pex cells. HIV was not visible in unexposed Pex cells (Fig. 6). However, negligible to low Pex cell autofluorescence was observed in the green channel for all coverslips examined (Fig. 6; see Fig. S5 in the supplemental material). Only external or internal HIV was visible in HIV-exposed Pex cells that were immunolabeled with the respective nonspecific goat or rabbit isotype control antibodies (Fig. S5).

10.1128/mbio.02177-21.4 FIG S4 HIV-1 can be internalized by Pex cells. The relative amount of intracellular HIV-1 was measured over 24 h following Pex cell exposure by using a double fluorometric immunolabeling technique to distinguish extracellular HIV from intracellular HIV, as described in the text. The percentage of intracellular HIV was then calculated and is shown on the *y* axis. Background levels of fluorescence were recorded for Pex cells not exposed to HIV (UE). HIV exposure resulted in HIV being found intracellularly, the level of which remained fairly stable over the time course assayed. The data shown represent the mean and variance of 3 assays performed in duplicate. *, *P* ≤ 0.0001 versus Pex cells not exposed to HIV. Download FIG S4, PDF file, 0.3 MB. Copyright © 2022 Day et al. 2022 Day et al. https://creativecommons.org/licenses/by/4.0/ This content is distributed under the terms of the Creative Commons Attribution 4.0 International license.

10.1128/mbio.02177-21.5 FIG S5 Assay controls for data shown in Fig. 6. Pex cells were incubated for 4 h with HIV-1 strain WITO before double immunolabel processing for confocal microscopy, as described in the text. (Right panel) External HIV-1 was immunolabeled using the goat polyclonal antibody LS-C103187, which recognizes HIV p17, p24, p53, p64, p120, and p160, and an Alexa Fluor 647 (AF647)-conjugated secondary antibody. Following permeabilization of host Pex cells, a rabbit IgG isotype control antibody and an AF488-conjugated secondary antibody were used to immunolabel internal/total virus. Internal virus was not recognized by the rabbit IgG control antibody, as indicated by the lack of a signal in the green channel. (Left panel) Pex cells were incubated with a goat IgG isotype control antibody and an AF647-conjugated secondary antibody. Pex cells were then permeabilized before incubation with the rabbit polyclonal antibody LS-C486994, which recognizes HIV p24, and an AF488-conjugated secondary antibody. External viruses were not recognized by the goat IgG control antibody, as indicated by the lack of a signal in the red channel. The data shown were obtained in parallel with data shown in Fig. 6 and demonstrate the specificity of the antibodies used to immunolabel both external and internal virus. For all coverslips examined, Alexa Fluor Plus 405-phalloidin was used to stain host Pex cells. Magnification, 63× with oil immersion. Download FIG S5, PDF file, 2.6 MB. Copyright © 2022 Day et al. 2022 Day et al. https://creativecommons.org/licenses/by/4.0/ This content is distributed under the terms of the Creative Commons Attribution 4.0 International license.

**FIG 7:**
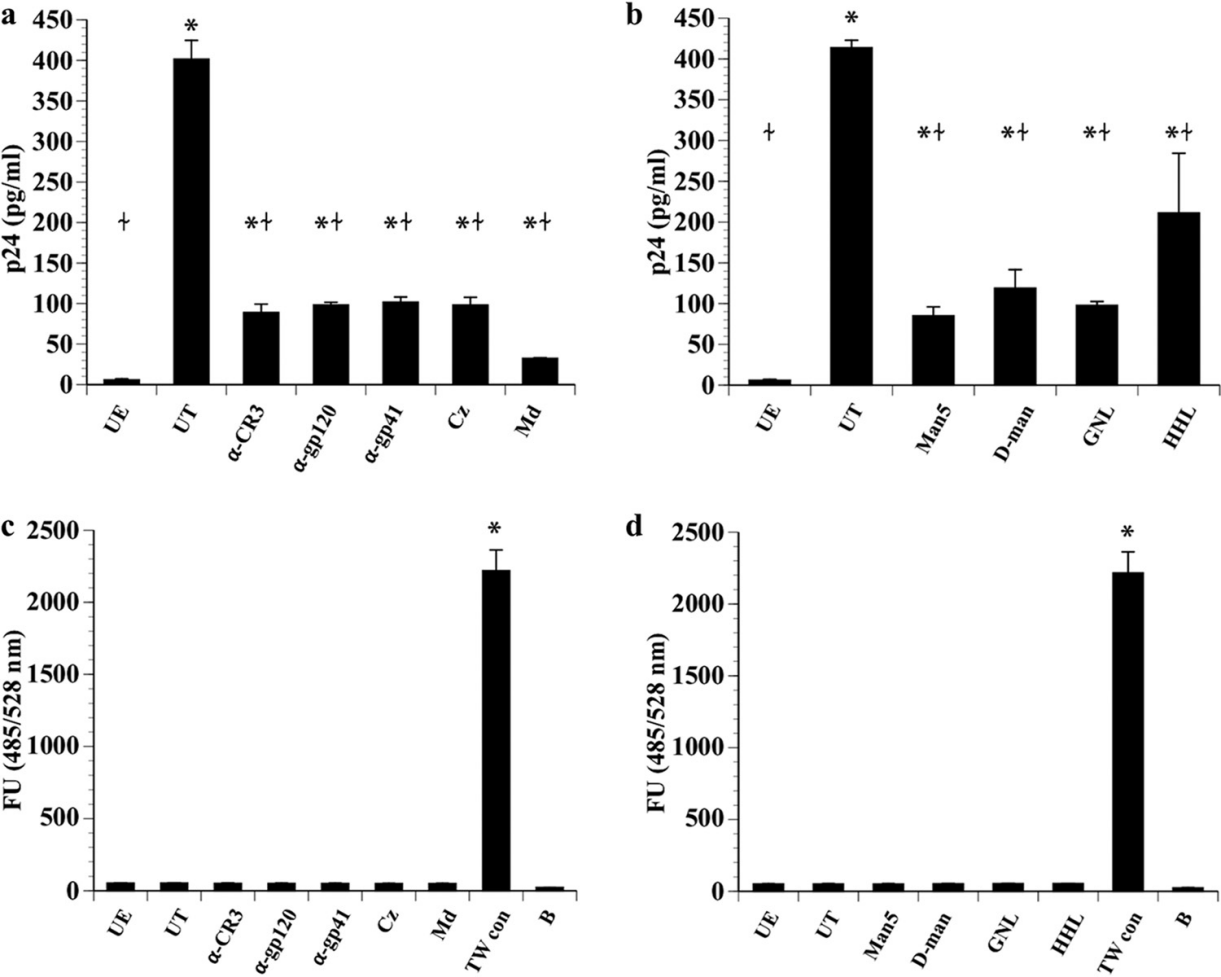
HIV-1 transcytosis across Pex cells is mediated by a CR3-Env interaction. HIV-1 strain WITO transcytosis across Pex cells in Transwell cultures was measured in the presence and absence of potential inhibitors of a CR3 I-domain–HIV envelope glycoprotein complex (a) or glycoprotein-associated mannose (b) interaction. HIV p24 in the basal Transwell chamber served as a readout for HIV transcytosis. FITC-dextran was added to the apical Transwell chamber with HIV and subsequently measured in the basal chamber medium, to ensure Pex cell integrity throughout each assay (c and d). (a) Antibody competimers tested included LM2/1 to CR3, A00019.01 to HIV gp120, or A00020.01 to HIV gp41. Carbamazepine (Cz) and methyldopa (Md), which bind to the CR3 I-domain with high affinity, were also tested. (b) The mannose glycans Man5 and α-d-mannopyranoside (d-man), as well as the mannose-binding lectins *Hippeastrum* hybrid lectin (HHL) and *G. nivalis* lectin (GNL), were tested. (c and d) To ensure Pex cell integrity throughout each assay, FITC-dextran was added to the apical Transwell chamber with HIV and subsequently measured in the basal chamber medium. Although a high level of fluorescence was recorded in the basal chamber of Transwells devoid of Pex cells (c and d [TW con]), only background fluorescence was recorded for competitive assays performed using Pex cells in the presence of antibodies or drugs (c) or when assays were performed with mannose glycans or mannose-binding lectins (d). Collectively, these data indicate that HIV-1 is able to transcytose across an intact Pex cell barrier by a mechanism involving gp120/gp41-linked mannose residues binding to the CR3 I-domain. The data shown represent the mean and variance of 3 assays performed in duplicate. UE, cells not exposed to HIV-1; UT, untreated—no blocking antibody, drug, glycan, or lectin was included in the assay; FU, fluorescence units; B, blank, medium control. *, *P* ≤ 0.0001 versus cells not exposed to HIV; ≁, *P* ≤ 0.0196 versus untreated cells.

We then performed a series of assays to determine whether the CR3-HIV interaction allowed for HIV transcytosis across an intact cervical epithelial cell barrier. Fluorescein isothiocyanate (FITC)-dextran was added with HIV-1 to polarized Pex cells in the upper chamber of a Transwell system to ensure membrane integrity was maintained throughout the course of each assay (5). Assays were first performed in the presence or absence of competimers with the potential to block the HIV-CR3 interaction (Fig. 7a). In the absence of any competimer, approximately 4% (401.41 ± 23.29 pg/mL) of the HIV inoculum (10 ng/mL p24 HIV equivalent) transcytosed across the Pex cell monolayer to the basal chamber. Transcytosis could be significantly (*P* ≤ 0.0001) impaired by the inclusion of antibodies to the CR3 I-domain or to HIV gp-120 or gp41, as well as by the CR3-blocking drug carbamazepine or α-methyldopa. Similar results were obtained when we repeated these experiments in the presence and absence of mannose glycan (Man5 and d-mannopyranoside) or mannose-binding lectin (*Hippeastrum* hybrid lectin [HHL] and Galanthus nivalis lectin [GNL]) competimers (Fig. 7b). A high level of fluorescence was recorded in the basal chamber medium of control Transwells that were devoid of Pex cells, which was not observed for Transwells containing Pex cells (Fig. 7c and d). Thus, Pex cells had retained their barrier function throughout the course of the assays.

Taken together, the above data indicate that the HIV-1 T/F strain WITO transcytosed across an intact Pex cell monolayer by a mechanism involving an interaction between CR3 and Env glycoprotein-linked mannose on the HIV surface.

### HIV-1 remains infective following transcytosis across primary cervical epithelial cells.

**FIG 8:**
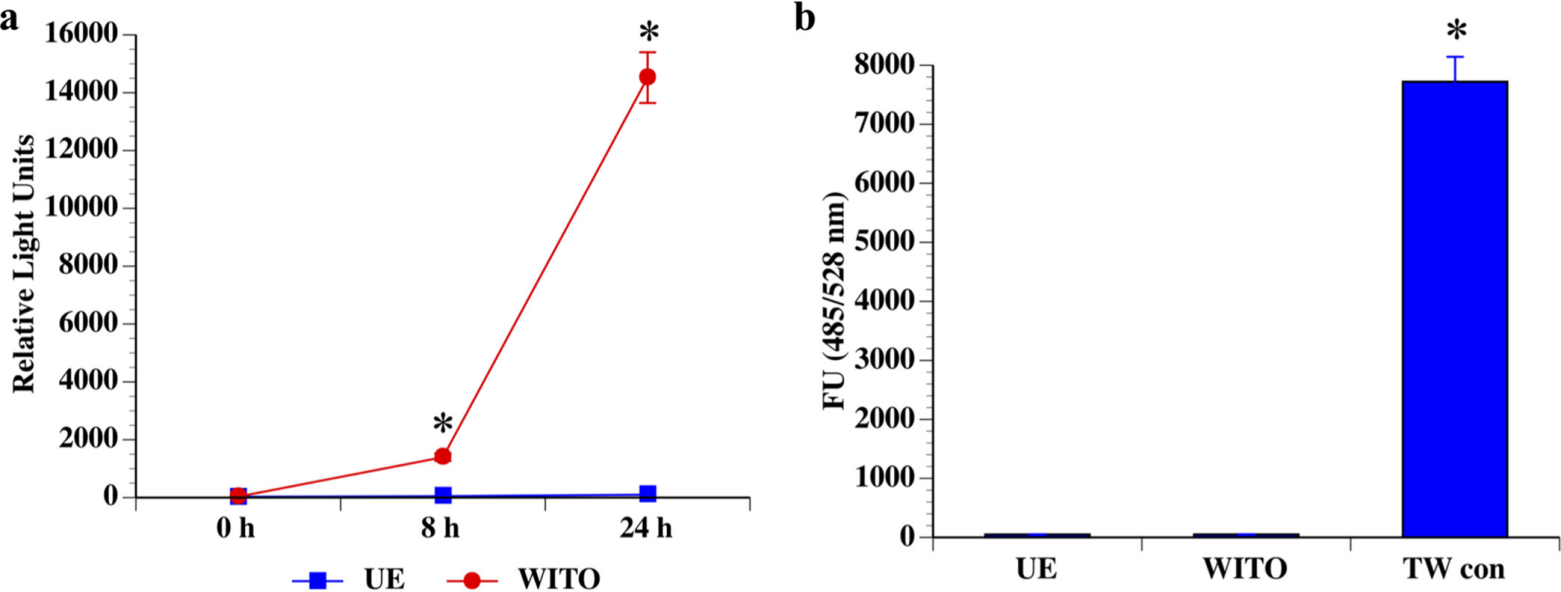
HIV-1 remains infective following transcytosis across Pex cells. (a) The infectivity of HIV-1 strain WITO was measured following its transcytosis across Pex cells by transferring the medium from the basal chamber of a Transwell transcytosis assay to TZM-bl reporter cells, as described in the text. Luminescence, indicative of HIV infection of TZM-bl cells, was recorded as relative light units (*y* axis) immediately upon infection (0 h) and at 8 and 24 h postinfection. (b) To ensure Pex cell integrity throughout each assay, FITC-dextran was added to the apical Transwell chamber with HIV during the initial transcytosis assay and subsequently measured in the basal chamber medium before its use to infect TZM-bl cells. The data shown represent the mean and variance of 3 assays performed in triplicate. UE, basal medium from transcytosis assays performed without HIV (control) (a); WITO, basal medium from transcytosis assays performed with HIV (a); FU, fluorescence units; UE, cells not exposed to HIV (b); TW con, blank Transwell devoid of Pex cells (b). *, *P* ≤ 0.0001 versus cells not exposed to HIV (a) or *P* ≤ 0.0001 versus Transwells containing Pex cells (b).

Successful infection of TZM-bl reporter cells by HIV induces the expression of luciferase, which serves as a well-established readout for HIV infectivity. Therefore, to examine whether HIV transcytosis across Pex cells resulted in infectious virus, rather than degraded or inactivated viral particles, we performed transcytosis assays in which medium from the basal Transwell chamber was harvested and then used to inoculate TZM-bl reporter cells to allow a synchronous exposure. Luminescence, indicative of HIV infection, was recorded in TZM-bl lysates at 0, 8, or 24 h postincubation (Fig. 8a). Only background levels of luminescence were recorded for TZM-bl cells that were not exposed to HIV-1 strain WITO. However, a significant (*P* ≤ 0.0001) increase in luminescence was observed between 8 h and 24 h for cells exposed to HIV. To ensure the integrity of the Pex cell monolayer during the initial HIV exposure, the potential presence of FITC-dextran in the basal medium was measured before its use to infect TZM-bl cells (Fig. 8b). As in previous assays, only background levels of fluorescence were observed in the presence of Pex cells, and a high level of fluorescence was recorded in control Transwells devoid of Pex cells. Thereby, strain WITO retained its infectivity following transcytosis across an intact Pex cell barrier.

## DISCUSSION

Young women are disproportionately affected by HIV infections. For example, the incidence of infection in women of age 15 to 24 years is 55% higher than that observed for men of the same age group (1, 62), and globally, HIV/AIDS continues to be the leading cause of death among women of reproductive age (63). Disruption (e.g., through physical abrasion) of the mucosal barrier that lines the FRT is thought to provide a portal through which HIV can access subepithelial immune cells, which in turn, mediates viral dissemination (5, 64, 65). Viral transcytosis through polarized (with tight junctions intact) epithelial cells represents an alternative mechanism of HIV transmission to subepithelial immune cells (12).

The immediate events that lead to HIV transmission through the uterine cervix are not fully understood. It is accepted that CD4 serves as the key host cell surface receptor for HIV fusion and entry into immune cells, including T-cells and monocytes (66, 67). Binding of HIV to CD4, followed by the engagement of a chemokine coreceptor (CCR5 or CXCR4 for R5- and X4-tropic HIV strains, respectively), initiates viral fusion with, and internalization into, CD4^+^ cells (68, 69). HIV also is able to bind to and to enter epithelial cells derived from the FRT (12, 70–74), which do not express CD4 (11, 12, 75, 76). Thereby, alternative host cell surface molecules most likely enable HIV transmission across FRT epithelia. In this regard, we have now provided the first evidence of a direct interaction occurring between HIV-1 and the CR3 I-domain (Fig. 1; Fig. S1 and S2). We further show that the CR3-HIV interaction mediates virus adherence to, and entry into, primary cervical epithelial cells (Fig. 3, 4, and 6), as well as virus transcytosis across an intact cervical epithelial barrier (Fig. 7 and 8).

Enhanced infection of complement-opsonized HIV via CR3 is shown for dendritic cells (19, 20, 22, 25), monocytes, and peripheral blood mononuclear cells (PBMCs) (21, 23, 24, 77). In contrast, using SPR, we found that a direct, high-affinity (approximate *K_d_* of 11 to 337 nM, depending on the isolate) interaction with CR3 occurred for each of the five (nonopsonized) PBMC-derived HIV-1 strains that we tested (Table 1). Myszka et al. (78) used isothermal titration calorimetry to reveal a *K_d_* value of 5 to 190 nM for binding of recombinant gp120 (i.e., Env) to CD4. Thus, the interaction of HIV with CR3 is of comparable binding affinity to that reported for the interaction between HIV gp120 and CD4.

We found that HIV adherence to CR3, and to the CR3 I-domain, was dependent upon a surface-exposed N-linked glycan on the virion surface, as pretreatment of virus with PNGase F eliminated HIV binding to CR3 and to the CR3 I-domain (Table 1). Nearly half of the mass of an HIV virion is comprised of carbohydrates (59). Env is heavily glycosylated—it contains an average of 93 N-linked glycans on each monomer of the mature trimeric protein (59). The binding affinity for HIV-1 WITO was dramatically (more than 35-fold) enhanced when this strain was propagated in PBMCs versus when it was derived from HEK293T cells, which are known to exhibit low levels of high-mannose glycosylations (79–81). Therefore, we suggest that high-mannose N-glycans on the HIV Env protein bind to the CR3 I-domain and, thereby, promote adherence to and transcytosis across primary cervical epithelial cells (Fig. 4, 6, and 7).

Productive, sustained, infection with HIV requires infection of CD4^+^ T cells. Within the FRT, this requires that HIV reach CD4^+^ immune cells within the subepithelial space. HIV may transcytose through genital epithelia to reach the lamina propria and, in turn, infect CD4^+^ T cells as cell-free virus. Alternatively, HIV may indirectly infect CD4^+^ cells via transinfection from subepithelial immune cells or fibroblasts that harbor infectious virus (6, 82–85). We found that cell-free virus was readily transcytosed across polarized Pex cell monolayers, and HIV remained infectious following Pex cell transcytosis (Fig. 7 and 8).

**FIG 9:**
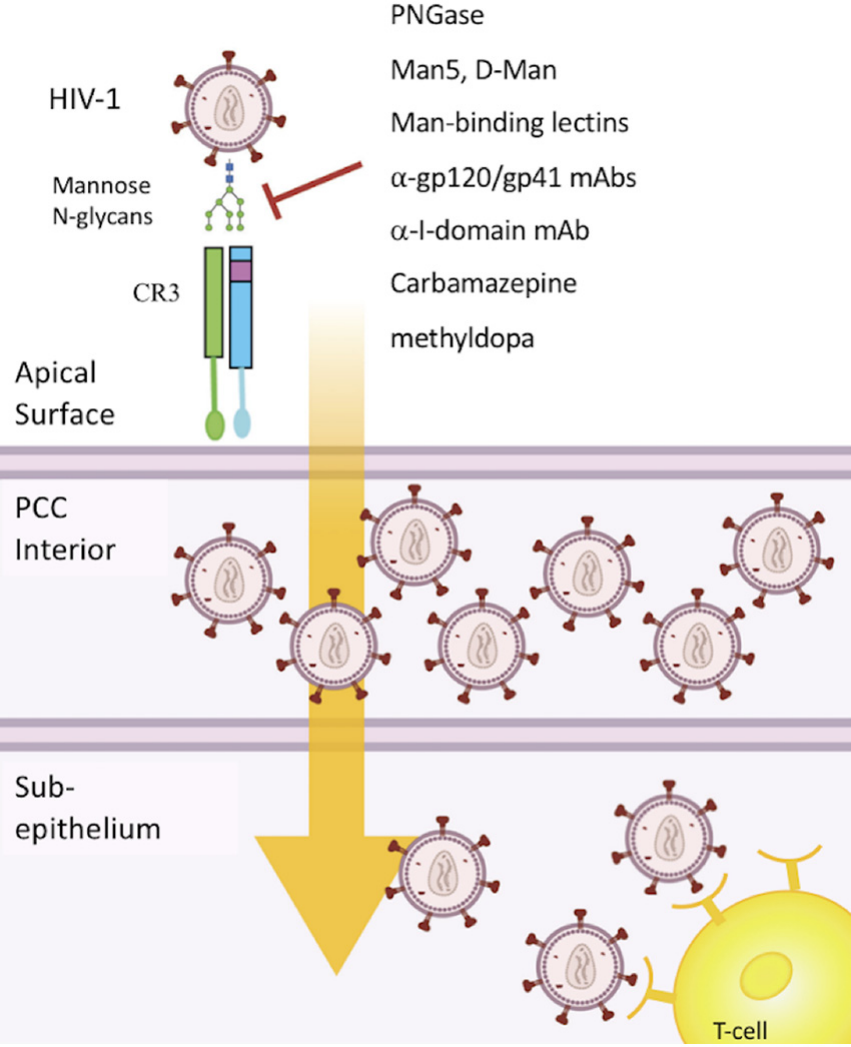
A working model of CR3-mediated HIV transmission through the cervix. The HIV-CR3 interaction may constitute an efficient pathway for HIV delivery to subepithelial lymphocytes following virus transmission across an intact cervical epithelial barrier. CR3 is highly expressed on the surface of the cervix. The I-domain region within CR3 serves as a multilectin binding site and exhibits high affinity for N-glycans present within the mannose (Man) patch of the HIV glycan shield. Removal of N-glycans by PNGase F treatment negates the HIV-CR3 (I-domain) interaction. Similarly, the interaction of HIV with Pex cells, is significantly impaired by mannose glycans, mannose-binding lectins, and small-molecule drugs that exhibit high affinity (*K_D_* of ∼2.12 nM for carbamazepine; *K_D_* of ∼1.01 nM for α-methyldopa) for the CR3 I-domain, as well as by antibodies to the CR3 I-domain or to the HIV Env glycoprotein complex (gp120/gp41). Engagement of CR3 by HIV triggers viral transcytosis across the intact cervical epithelial barrier with the subsequent delivery of virus to lymphocytes, including CD4^+^ T cells, which reside in high numbers within the subepithelial space of the cervix (84, 87). Thus, CR3 may play a unique role on epithelial cells in dually facilitating HIV-1 attachment and transmission. This model is consistent with the different cellular effects reported for CR3 engagement, which are dependent upon the specific CR3-ligand interaction (32, 35–37). The figure shown was created using BioRender.com. PCC, primary cervical cell.

CR3 is highly expressed on the ecto- and endocervix, limited or no expression occurs on the endometrium and fallopian tube epithelia, and no expression occurs on the vaginal epithelium (18). The predominant expression of CR3 on the luminal surface of the cervix may have important implications related to HIV transmission in that the presence of CD4-expressing cells within the subepithelia of the FRT exhibits similar compartmentalization. That is, the highest concentration of CD4-expressing cells within the FRT is found within the lamina propria of the cervical transformation zone and ectocervix, whereas CD4^+^ cells are sparsely distributed within the subepithelium of the vagina (86, 87). Therefore, CR3-mediated transcytosis through the cervical epithelium would result in the juxtaposition of HIV with CD4-expressing cells within the subepithelial space (Fig. 9). Thus, our data indicating a role for CR3 in mediating viral transmission across the cervical epithelium is consistent with the idea that the cervical epithelium serves as the primary site for HIV transmission across the FRT. These data are also consistent with the observation that gonococcal infection promotes susceptibility to HIV (88) in that the upregulation of CR3 on the Pex cell surface (56) during gonococcal infection may provide one explanation for this observation. Indeed, we found a strong positive correlation existed between the surface expression of CR3 on Pex cells and HIV adherence, which was enhanced in the presence of NgPLD (Fig. 5).

Collectively, our data support a role for CR3 in HIV transcytosis of polarized cervical cells, but they do not negate the possibility that CR3-mediated HIV entry and transcytosis across Pex cell monolayers might be augmented by a coreceptor. That is, HIV transcytosis across Pex cells could be dramatically reduced (∼75%), but not completely abolished, by the presence of antibodies, drugs, or molecules with the potential to block a CR3-HIV interaction (Fig. 7). CR3 is known to serve as an important signal transducer in professional phagocytic cells through its ability to interact with several proteins. In this regard, CR3 mediates intracellular signaling events by proteins that lack transmembrane and cytoplasmic sequences (e.g., glycosylphosphatidylinositol [GPI]-linked proteins) and, thus, would independently have no other means of signal initiation (89–94). Transmembrane (e.g., FcγRII, Toll-like receptors, cell adhesion molecules) and soluble (e.g., FcγRII and FcγRIIIB) proteins can also form coreceptor complexes with CR3 that lead to a variety of cellular functions (91, 93, 94). Notwithstanding, an alternative explanation for our inability to completely prohibit HIV transcytosis by blocking the CR3-HIV interaction is that it simply might be technically challenging to completely prohibit the HIV-CR3 interaction in a cell culture model by using an antibody, or a drug, directed against the I-domain or against HIV-linked mannose. CR3 follows an endocytic recycling pathway (95–98), as well as oscillating between active and nonactive conformations on the host cell surface (30–34). Similarly, the Env spike protein can transiently shift between three distinct conformations, which dictate binding (or not) to host target molecules (99). HIV neutralizing antibodies can lock Env in a conformation that prevents viral infection (99). Additionally, whereas ICAM-1 (which binds to the CR3 I-domain and can serve as a CR3 coreceptor) (91, 93, 94) incorporation into the viral envelope with budding is thought to enhance LFA-1 (integrin α_L_β_2_, CD11a/CD18)-mediated HIV-1 infection of CD4^+^ T cells (100), the potential effect of ICAM-1 binding to the CR3 (integrin α_M_β_2_, CD11b/CD18) I-domain on Pex cells is not known. Free ICAM-1, or ICAM-1 within the context of the HIV envelope, could (i) reduce the efficiency of the competimers tested with regard to their ability to bind to the I-domain during competitive adherence or transcytosis assays, (ii) trigger a CR3-dependent signaling pathway within Pex cells that is not conducive to HIV uptake or its transcytosis, and/or (iii) alter the conformation of CR3 and/or Env in a manner that does not promote the CR3-HIV interaction. These possibilities may not be mutually exclusive in that the dynamics of the HIV-CR3 interaction are likely complex.

Several host cell surface (receptor) molecules can promote the adherence of HIV-1 to CD4-negative cells, including galactosylceramide (GalCer), heparan sulfate, and the mannose receptor (6, 14, 16, 101, 102). On CD4^+^ cells, numerous cell surface molecules have been identified that may promote HIV infection via an interaction with gp120/gp41, including DC-SIGN, the mannose receptor, langerin, CXCR6, and CCR8 (103), none of which are known to be expressed by cervical epithelial cells (15, 104). Whereas HIV uses the canonical coreceptor CXCR4 or CCR5 for entry into CD4^+^ cells, whether FRT epithelial cells express CXCR4 or CCR5 and, if expressed, the extent to which HIV has affinity for these receptors in the absence of CD4 are controversial (105). CXCR4 and CCR5 (as well as CR3 [18]) are not expressed by the ectocervical and endocervical epithelial cell lines Ect1 and End1, respectively (72). However, CXCR4 and CCR5 appear to be expressed by primary genital epithelial cells obtained from vaginal swab scrapings (6). Blocking CXCR4 and/or CCR5 significantly reduces HIV-1 entry and transcytosis of these cells but does not block the initial adherence of HIV (6). Thus, our finding that blocking CR3 not only inhibited HIV adherence but also substantively reduced HIV entry and transcytosis of Pex cells reveals a new and unique role for CR3 in dually facilitating HIV attachment and entry into cervical epithelial cells.

The constitutive expression of CR3 on the human cervical surface appears to be unique and may be limited to humans and to great apes (106). CR3 is not expressed within the murine female genital tract (106), nor have we been able to detect CR3 in cervical biopsy specimens obtained from rhesus macaques. Moreover, the lectin function of the human CR3 I-domain may not be extended to mice (54). These observations further complicate the already technically challenging field of animal model development for HIV research. Similarly, to date, CR3 has not been described to be present or functional on other human mucosal surfaces. Hussain et al. (107) used monoclonal antibodies to CD11b and CD18 to examine the presence of CR3 in rectal tissue by immunohistochemistry. Of the specimens examined, approximately 50% expressed both CR3 subunits on a subset of surface and crypt rectal epithelial cells. However, whether CR3 is functional on the rectal epithelium has not been examined. Thereby, an understanding of the mechanism driving HIV transcytosis through the cervix, and the role that CR3 plays in directing the intracellular migration of HIV, may have broad implications and will be the focus of future studies.

## MATERIALS AND METHODS

### Cell culture.

Primary cervical epithelial (i.e., Pex) cells were procured from surgical cervical tissue and maintained in defined keratinocyte serum-free medium (dk-SFM) (Gibco, Grand Island, NY, USA), as described elsewhere (108). Deidentified, healthy, cervical tissues were obtained from premenopausal women and were provided by the Human Tissue Resource Network/Cooperative Human Tissue Network (Columbus, OH). Over the past 20 years, we have not found any significant variability (in the parameters that we have examined) among Pex cells derived from thousands of tissue donors.

CHO cells (i.e., CHO-neo, the vector control parent cells, and CHO-CR3, CR3-expressing cells [109]) were a gift from R. Ingalls (Boston University, Boston, MA, USA) and from L. Schlesinger (Texas Biomedical Research Institute, San Antonio, TX, USA). CHO cells were maintained in Ham’s F-12 medium (Gibco) supplemented with 5% fetal bovine serum (FBS) (Gibco) plus 0.5 mg/mL G418 (Gibco).

TZM-bl cells, the HIV reporter (catalog no. 8129), were obtained through the NIH AIDS Reagent Program, Division of AIDS, NIAID, NIH, from John C. Kappes and Xiaoyun Wu (110–114). TZM-bl reporter cells are a HeLa cell derivative that stably express high levels of known HIV receptors (CD4, CXCR4, and CCR5) on their cell surface (112). These cells also express luciferase (for R5-tropic HIV) and β-galactosidase (for X4-tropic HIV) reporter genes, each of which is under transcriptional control of the HIV-1 long terminal repeat (110, 111, 114). TZM-bl cells were cultured in Dulbecco’s modified Eagle’s medium (DMEM) (Gibco) supplemented with 5% FBS. TZM-bl cells are a well-established model by which to measure HIV infectivity.

### HIV-1 propagation.

Plasmid DNA containing full-length HIV proviral DNA was obtained from the NIH AIDS Reagent Program for the following HIV-1 strains: NL4-3 (from Malcolm Martin; catalog no. 114) (115), NL4-3 AD8 (from Eric O. Freed; catalog no. 11346) (116), REJO (catalog no. 11746), RHPA (catalog no. 11744), and WITO (catalog no. 11739) (the latter all from John Kappes and Christina Ochsenbauer) (117–121). Primary cell-derived virus particles were produced from phytohemagglutinin-activated PBMCs, as previously described (122, 123). Cell-line-derived virus particles were produced by transfection of proviral DNA into HEK293T cells, also as described previously (122). GFP-tagged HIV particles were generated by cotransfecting GFP-Vpr (NIH AIDS Reagent Program, from Thomas J. Hope; catalog no. 12482) with the indicated full-length proviral DNA (124). Virions from all cell types were purified by ultracentrifugation through a sucrose cushion, and the quantity and infectivity of virus were measured by HIV p24 protein enzyme-linked immunosorbent assay (ELISA) (Xpress Bio, Frederick, MD) and by infection of TZM-bl reporter cells, as described previously (122, 123).

### Surface plasmon resonance analyses of the interaction of HIV with CR3 and the CR3 I-domain.

The interaction of HIV-1 strains with human rI-domain (expressed and purified using a published method [54]) and rCR3 (R&D Systems, Minneapolis, MN) was analyzed by SPR using a Biacore S200 system (Cytiva [formerly GE Healthcare Life Sciences], Parramatta, NSW, Australia). A blank immobilization control/reference flow cell of ethanolamine was used on all chips. Analyses of human rCR3 and rI-domain with Man5 glycan [Manα1-3(Manα1-6)Manα1-3(Manα1-6)Man] and/or HIV-1 were performed as previously outlined by Poole et al. (54). Where noted, HIV strains were pretreated with PNGase F (New England Biolabs, Ipswich, MA) to remove N-linked glycans before being immobilized onto a series S C1 sensor chip to a minimum capture level of 600 response units (RU). For competitive SPR assays, competition between Man5 or drugs (carbamazepine or α-methyldopa) and rCR3 and/or rI-domain at 1 μM concentration was tested. I-domain, Man5, or drug, as well as I-domain preincubated with Man5 or drug, was injected over immobilized HIV-1 strain WITO, which had been generated in primary human PBMCs. SPR sensorgrams and result plots for all experiments were analyzed using Biacore S200 evaluation software (Cytiva).

### Fluorometric HIV adherence and CR3 expression assays.

Fluorometric adherence assays were performed essentially as described by Jen et al. (125), with modification as outlined below. GFP-tagged HIV-1 strains NL4-3 or NL4-3 AD8 were propagated in HEK293T cells and then fixed in 4% paraformaldehyde. Relative viral adherence was then determined by incubating 10 ng/mL (p24 HIV equivalent) of each strain with Pex or CHO cell monolayers in 96-well plates for 2 h in the presence or absence of 100-fold serial dilutions (100 μM to 100 pM) of carbamazepine (Selleck Chemicals, Houston, TX), which served as a competitive inhibitor of a potential HIV-1–CR3 I-domain interaction (54). Dimethyl sulfoxide (DMSO; 1%) served as a vehicle control. Inoculated (no drug, with vehicle) and uninoculated (with drug or vehicle) control cell assays were treated in parallel with competitive drug inhibition assays.

To quantify HIV adherence, Pex or CHO cells in 96-well plates were inoculated with HIV-1 strain NL4-3 AD8 or WITO (10 ng/mL of p24 HIV equivalent). Where indicated, Pex cells or HIV-1 strain WITO were preincubated (30 min) with (mouse) antibodies (LM2/1, specific to the CR3 I-domain; E-11, specific to CD35 [control]; A00019.01 specific for HIV gp-120; or A00020.01, specific for gp41; all from Santa Cruz Biotechnology, Dallas, TX, and used at 10 ng/mL) with the potential to block adherence mediated by an HIV-CR3 I-domain interaction. Alternatively, and as noted, experiments were performed using clarified, primed medium that either contained or did not contain NgPLD. Primed medium was generated by harvesting the medium from a 6-h infection of cycloheximide-treated Pex cells with NgPLD-expressing N. gonorrhoeae strain 1291 or a 1291 *pld* deletion mutant (control), as described previously (126). In brief, gonococci were removed from harvested medium by centrifugation and by filtration of the supernatant through a 0.22-μm-pore low-protein-binding syringe filter to produce wild-type or *pld*-mutant “primed” supernatants. Primed medium was then sequentially centrifuged through Amicon centrifugal filter units (Millipore Corporation, Bedford, MA) possessing molecular weight cutoffs (MWCO) of greater than 100 kDa, 30 kDa, and 50 kDa. A 1/10 volume of the filter-retained, secreted gonococcal products was then added to Transwell cultures, as indicated, to yield a 1× final solution of NgPLD (or control supernatant) equivalent to that produced by *N. gonorrrhoeae* during a Pex cell infection. As an enzyme, NgPLD activity is not linear. Thus, an NgPLD dose response was not examined.

Following incubation for the noted times, cells were fixed with 2% paraformaldehyde, and HIV was then immunolabeled (overnight [o/n] with rotation at 4°C) using the goat polyclonal antibody LS-C103187 (Lifespan Biosciences, Seattle, WA), followed by a 1-h (room temperature [RT]) incubation with a fluorescein isothiocyanate (FITC)-conjugated secondary antibody (Jackson ImmunoResearch Laboratories, West Grove, PA). Antibody LS-C103187 recognizes all of the major HIV-1 proteins, including p17, p24, p53, p64, gp120, and gp160. The fluorescence units recorded were adjusted for background, and HIV adherence was quantified using a standard curve derived from 2-fold serial dilutions of NL4-3 AD8 or WITO (from 10 ng to 0 ng of p24 HIV equivalent). In parallel, and where noted, the relative expression of CR3 on the host cell surface was similarly determined, as we have described previously (55), in which separate wells of the microtiter plate were immunolabeled using the mouse anti-CR3 antibody VIM12 (Santa Crus Biotechnology, Santa Cruz, CA), which is specific for the CR3 lectin domain within CD11b, and a FITC-conjugated secondary antibody. CR3 was recorded as relative fluorescence units and adjusted for background.

For immunolabeling experiments, omission of the primary and secondary antibodies served as negative controls for nonspecific labeling and autofluorescence. Blank wells, devoid of Pex or CHO cells but that were inoculated with virus, served as a control for nonspecific binding for all adherence assays. Fluorescence (485/528 nm) intensity, corresponding to viral adherence or to CR3 surface expression, was recorded using a Synergy HT multimode microplate reader (BioTek Instruments, Winooski, VT, USA). Each assay was performed in triplicate on 3 separate occasions.

### Determination of intracellular HIV-1.

The relative percentage of internal versus external HIV was measured over the course of a 24-h exposure. Pex cells in 24-well plates were inoculated with 10 ng/mL (p24 equivalent) WITO HIV-1. At the noted times postinoculation, the cells were fixed with 2% paraformaldehyde and subsequently immunolabeled (o/n with rotation at 4°C) with goat anti-HIV antibody LS-C103187 and an anti-goat peroxidase-conjugated secondary antibody (1 h at RT with rotation). Cells were then permeabilized (20 min with 0.2% Triton X-100) before the addition of the mouse Alexa Fluor 488-conjugated antibody 24-4 (Santa Cruz), specific to HIV p24 (o/n with rotation at 4°C). Cells were harvested in 250 μL QuantaBlu fluorogenic peroxidase substrate (Pierce Biotechnology, Rockford, IL) by scraping, and 100 μL of the cell lysate was transferred to each of 2 wells of a 96-well plate. Fluorescence intensities, corresponding to external (excitation, 320 nm; emission, 420 nm) or total (excitation, 485 nm; emission, 528 nm) HIV, were dually recorded using a Synergy HT multimode microplate reader. Fluorescence units recorded were adjusted for background, and HIV was quantified using a standard curve (as described above). The percentage of internalized HIV was then determined as [(total HIV − external HIV)/total HIV] × 100. Omission of each primary antibody, as well as unchallenged Pex cells, served as negative controls for nonspecific labeling and autofluorescence. Each assay was performed in duplicate on 3 separate occasions.

As a second approach, HIV internalization was qualitatively examined via confocal microscopy. Pex cells were grown to confluence on placental collagen-coated coverslips before a 4-h incubation with HIV-1 strain WITO. Where noted, Pex cells and virus were, respectively, preincubated (30 min) with mouse antibodies (10 ng/mL) to the CR3 I-domain (LM2/1) or to HIV gp120 (A00019.01). Antibody LS-C103187, followed by a donkey anti-goat Alexa Fluor 647-conjugated secondary antibody (Invitrogen, Waltham, MA), was used to label external virus. Cells were then permeabilized (at RT, with rotation) for 15 min using 0.2% Triton X-100 (Sigma-Aldrich, St. Louis, MO). Postpermeabilization, rabbit polyclonal antibody LS-C486994 (Lifespan Biosciences; specific for p24) and an alpaca anti-rabbit AF488-conjugated secondary antibody (Invitrogen) were used to immunolabel internal/total virus. Cells were then stained, per the manufacturer’s instructions, with Alexa Fluor Plus 405-phalloidin (Invitrogen) for 30 min. Controls included the following: Pex cells not exposed to HIV and the omission of the primary antibody from the immunolabeling procedure, as well as the use of rabbit and goat IgG isotype primary antibodies (Invitrogen) in the immunolabeling procedure. Labeled coverslips were viewed using a Zeiss 800 confocal microscope located at the Research Institute at Nationwide Children’s Hospital (Columbus, OH).

### Transcytosis assays.

Transcytosis assays were performed using the T/F strain WITO (propagated in HEK293T cells). Competitive assays were performed in which agents with the potential to block an HIV-CR3 I-domain interaction were included or omitted, and comprised antibodies (10 ng) to the CR3 I-domain (LM2/1), HIV gp120 (A00019.01), and HIV gp-41 (A00020.01), the I-domain-binding drugs (10 μM) carbamazepine and α-methyldopa, the glycans (10 μM) Man5 and methyl α-d-mannopyranoside (Selleck Chemicals), or the mannose-binding lectins (10 μg/mL) *Hippeastrum* hybrid lectin (HHL) and Galanthus nivalis lectin (GNL) (both purchased from Vector Laboratories; Burlingame, CA).

For all assays, Pex cells were grown to confluence in Falcon, 0.4-μm pore size, Transwell inserts (Corning Life Sciences, Tewksbury, MA) suspended in 24-well, non-tissue culture-treated plates. Cellular polarity was determined using an EVOM^2^ epithelial voltohmmeter (World Precision Instruments, Sarasota, FL) and defined as an electrical resistance greater than 3,000 Ω/cm^2^ as measured across the Pex cell monolayer. To ensure that Pex cell polarity was maintained throughout the course of each assay, FITC-dextran (1 mg/mL) was added to the apical chamber with HIV. A blank Transwell, devoid of Pex cells and to which HIV was not added, served as a control. Aliquots (100 μL) of medium from the basal chamber were transferred to each of 2 wells of a 96-well plate. Fluorescence (excitation, 485 nm; emission, 528 nm) intensity was then measured using a Synergy HT hybrid multimode microplate reader (BioTek Instruments), wherein increased fluorescence corresponded to a loss of membrane integrity. Two additional (100 μL) aliquots were used to measure HIV p24 in the basal chamber using the HIV-1 p24 ELISA kit (Xpress Bio, Frederick, MD) according to the manufacturer’s instructions. Absorbance was measured at 450 nm using a Synergy HT hybrid multimode microplate reader (BioTek Instruments). The concentration of p24 in each sample was determined by plotting data obtained against a p24 standard curve after adjusting for background. Experiments were performed in duplicate on 3 separate occasions.

### HIV infectivity assay.

To determine whether HIV-1 retained its infectivity following transcytosis through Pex cells, transcytosis assays were performed using the T/F strain WITO, as described above. Following a 48-h incubation, medium in the basal chamber was collected, and Pex cell membrane integrity was fluorometrically assessed, also as outlined above. Medium was then used to inoculate TZM-bl HIV reporter cell monolayers in a separate 24-well plate. TZM-bl monolayers were processed immediately following HIV exposure and at 8 and 24 h postexposure. In this regard, at each time point, the culture medium was removed, and the cells were extensively washed with phosphate-buffered saline. The cells were then lysed in Pierce lysis buffer (Thermo Scientific, Waltham, MA) by vigorous scraping, followed by a 15-min incubation (at RT with shaking). Cell lysates were collected and stored at −80°C. Samples for all time points were thawed (allowing for one freeze-thaw cycle to ensure lysis) and centrifuged (12,000 × *g* for 15 s), and triplicate aliquots (20 μL) of the resulting supernatant were transferred to a 96-well plate. Uninfected TZM-bl monolayers served as assay controls and were processed in parallel with HIV-infected cells. Luciferase activity was determined using the Promega luciferase assay system (Promega, Madison, WI), according to the manufacturer’s instructions. Relative light units, indicative of HIV-induced luciferase activity, and thus, HIV infection of TZM-bl cells, were recorded using a Synergy H1 hybrid multimode microplate reader (BioTek). Assays were performed on 3 separate occasions.

### Statistical analyses.

Statistical significance of data obtained was determined using a nonparametric analysis of variance (ANOVA). A Spearman’s correlation was used to assess potential associations between CR3 expression and HIV adherence, with and without the presence of NgPLD.
