# Supplementary material for: Complement Receptor 3 Mediates HIV-1 Transcytosis across an Intact Cervical Epithelial Cell Barrier: New Insight into HIV Transmission in Women
Source: mBio. 2022 Jan 11;13(1):e02177-21. doi: 10.1128/mbio.02177-21 (PMC8749410; doi:10.1128/mbio.02177-21)
Supplement: FIG S1 [file mbio.02177-21-sf001.pdf]

A) NL4-3 AD8 produced in HEK293T immortalized kidney cells interacting with the human rI-domain.

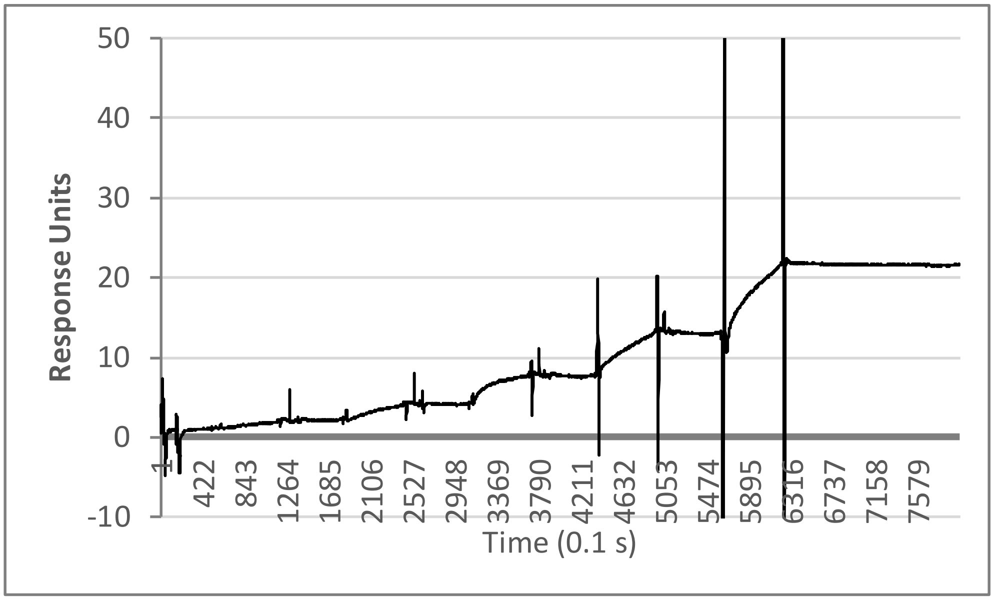

(B) PNGase F-treated NL4-3 AD8 produced in HEK293T immortalized kidney cells interacting with the human rI-domain.

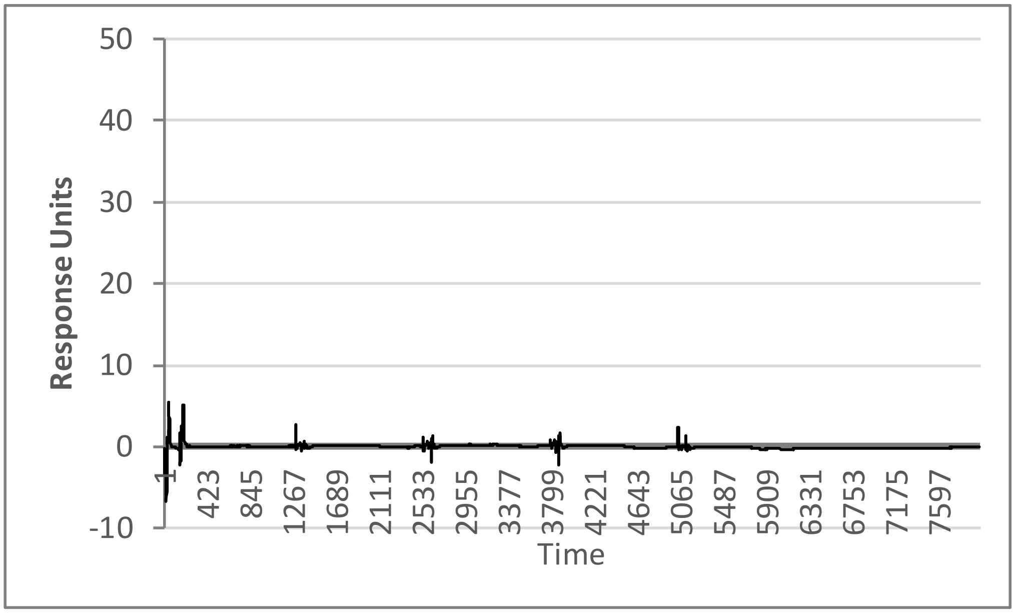

(C) NL4-3 AD8 produced in HEK293T immortalized kidney cells interacting with the human rCR3.

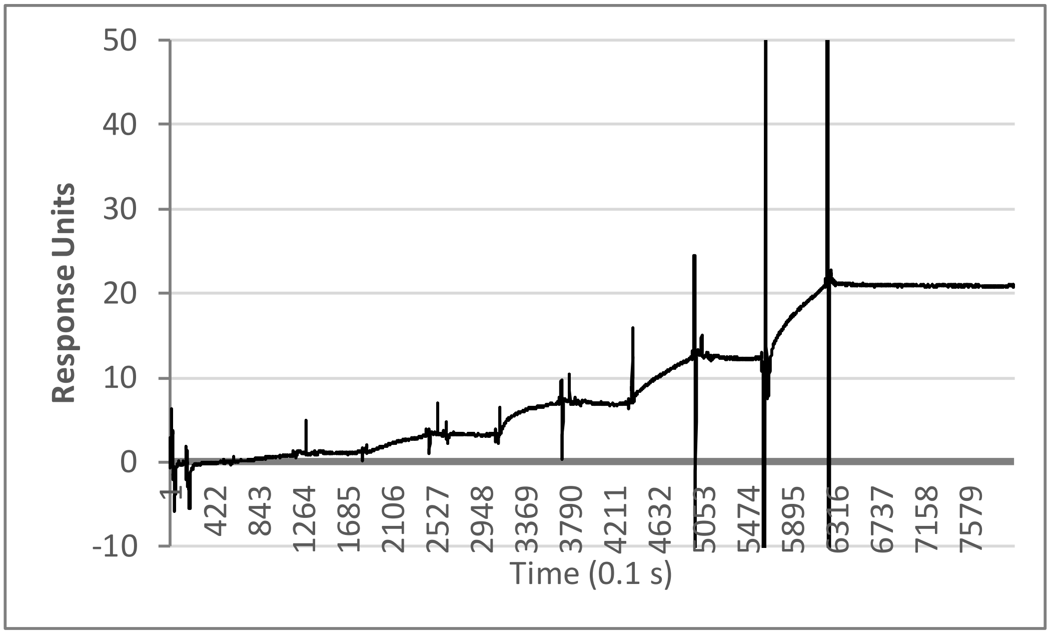

(D) PNGase F-treated NL4-3 AD8 produced in HEK293T immortalized kidney cells interacting with the human rCR3.

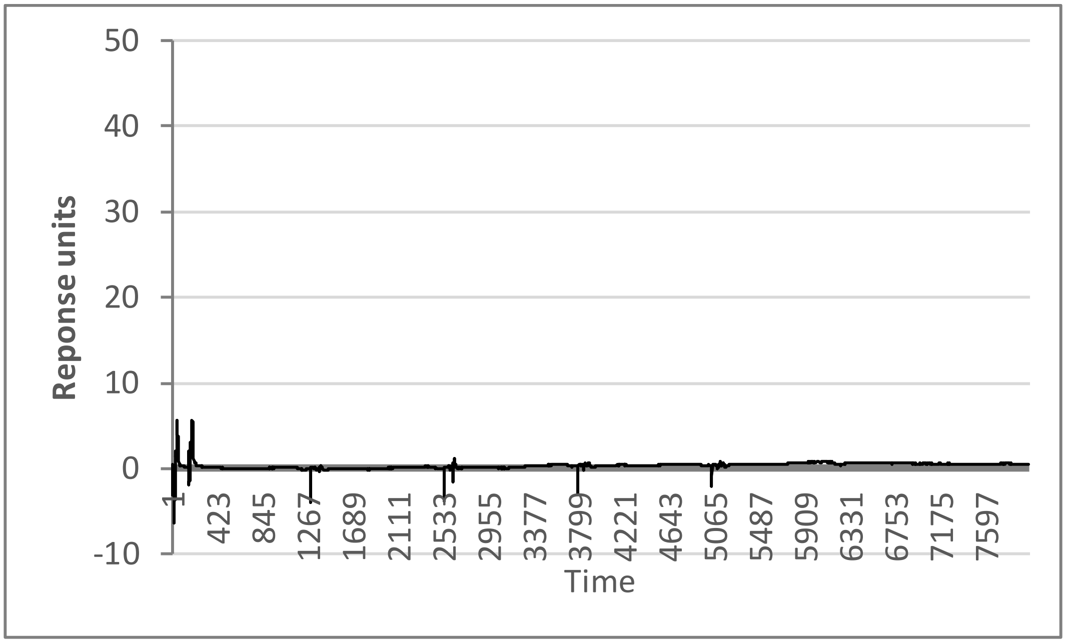

(E) NL4-3 produced in HEK293T immortalized kidney cells interacting with the human rI-domain.

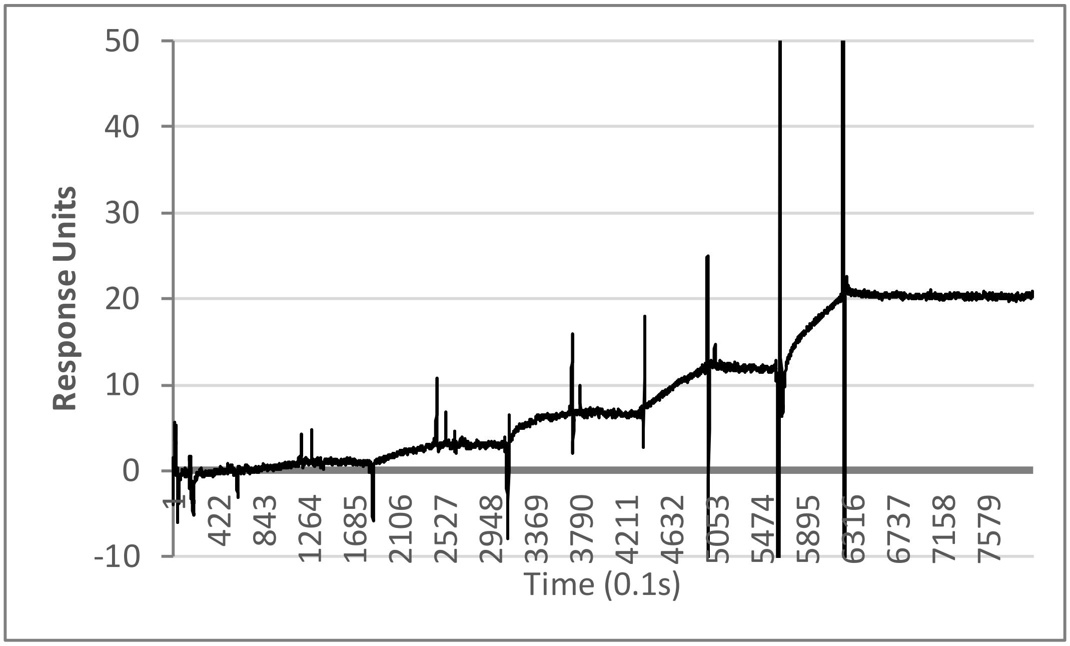

(F) PNGase F-treated NL4-3 produced in HEK293T immortalized kidney cells interacting with the human rI-domain.

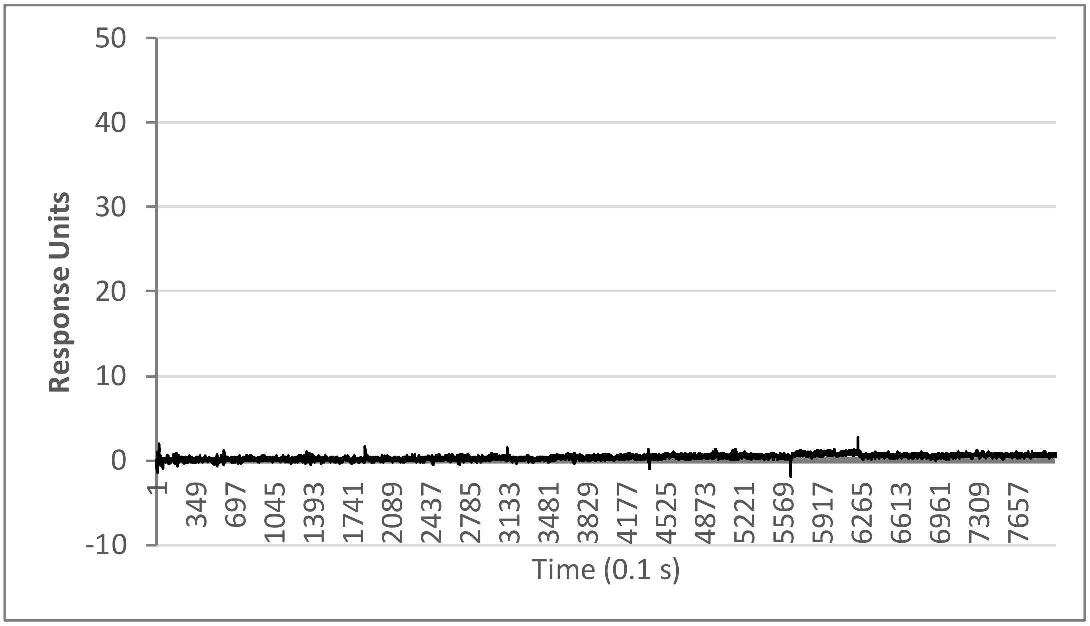

(G) NL4-3 produced in HEK293T immortalized kidney cells interacting with the human rCR3.

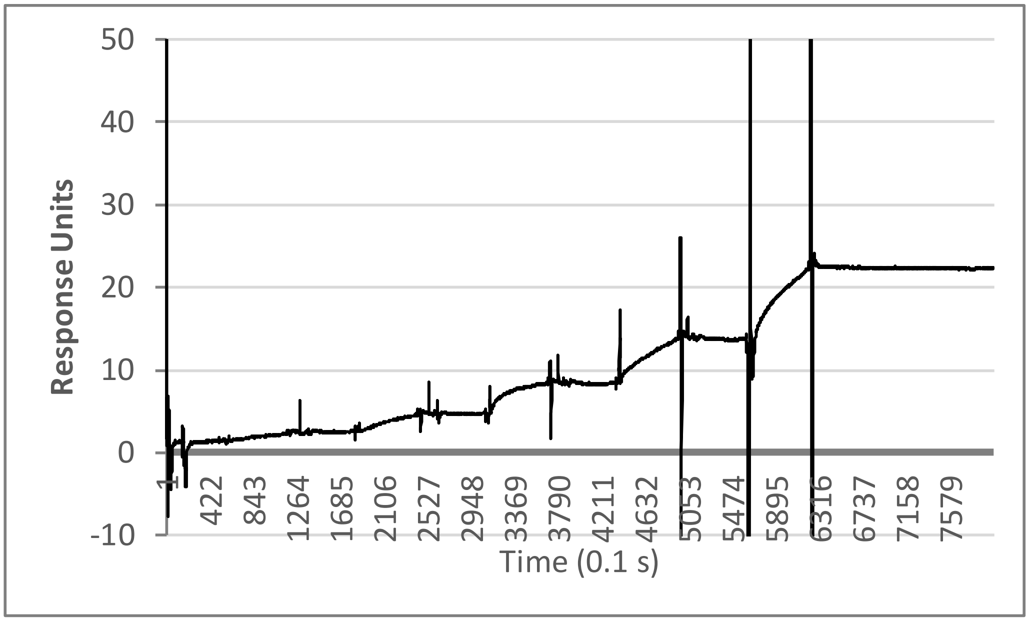

(H) PNGase F-treated NL4-3 produced in HEK293T immortalized kidney cells interacting with the human rCR3.

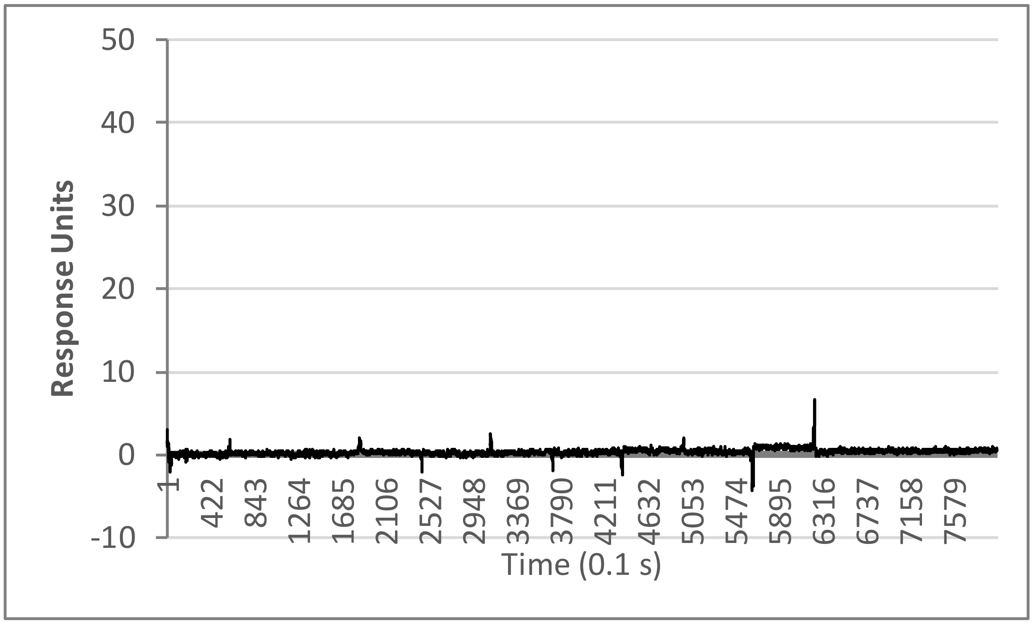

(I) RPHA produced in HEK293T immortalized kidney cells interacting with the human rI-domain.

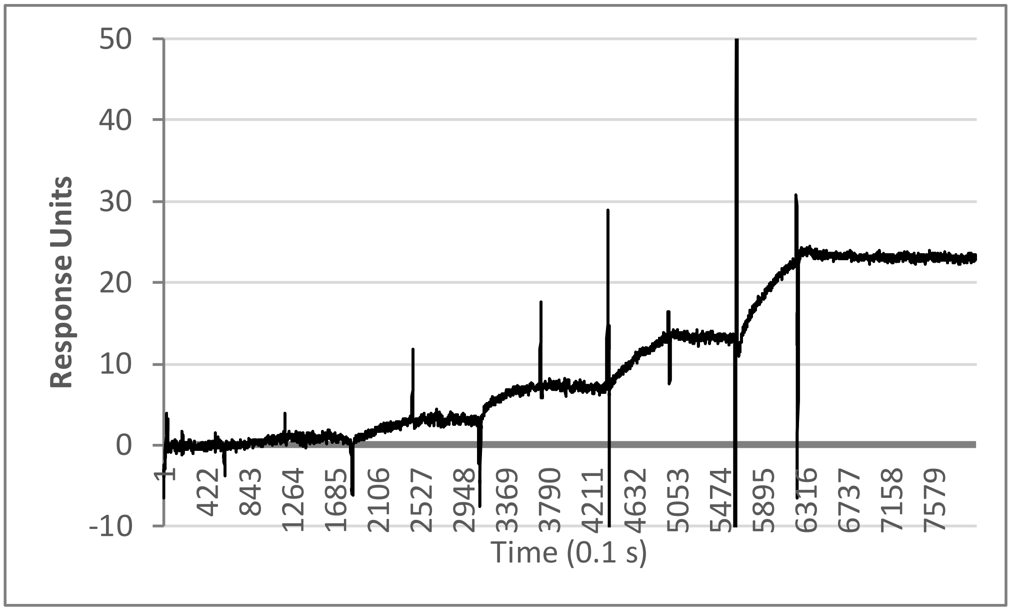

(J) PNGase F-treated RPHA produced in HEK293T immortalized kidney cells interacting with the human rI-domain.

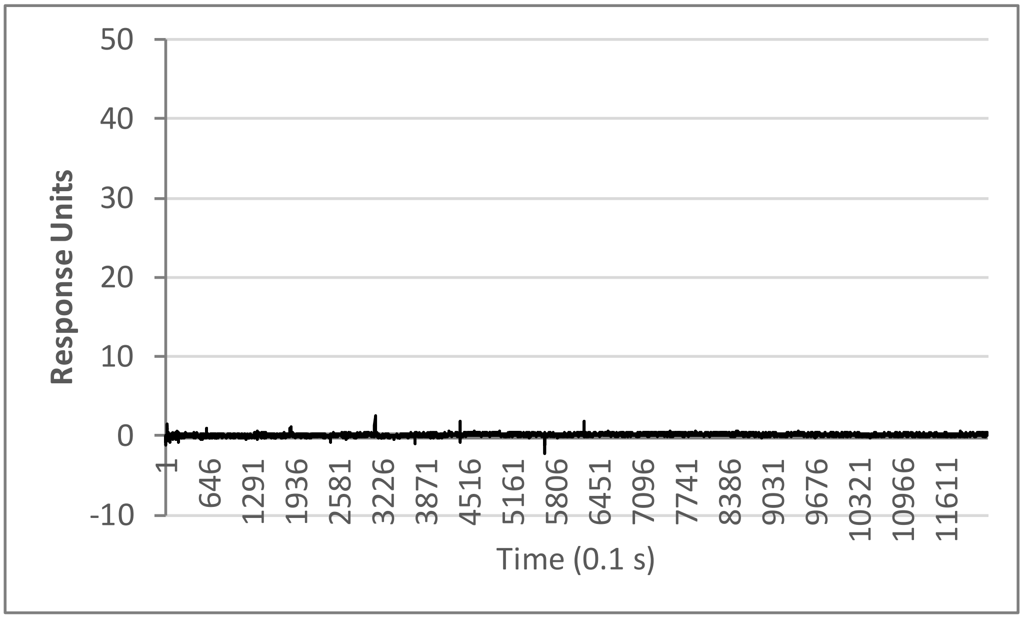

(K) RPHA produced in HEK293T immortalized kidney cells interacting with the human rCR3.

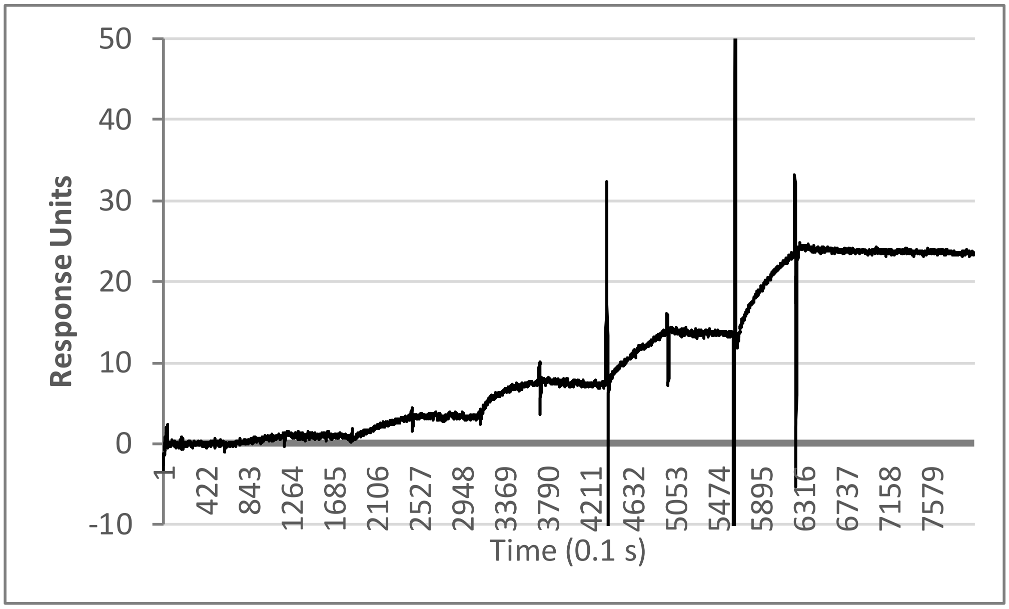

(L) PNGase F-treated RPHA produced in HEK293T immortalized kidney cells interacting with the human rCR3.

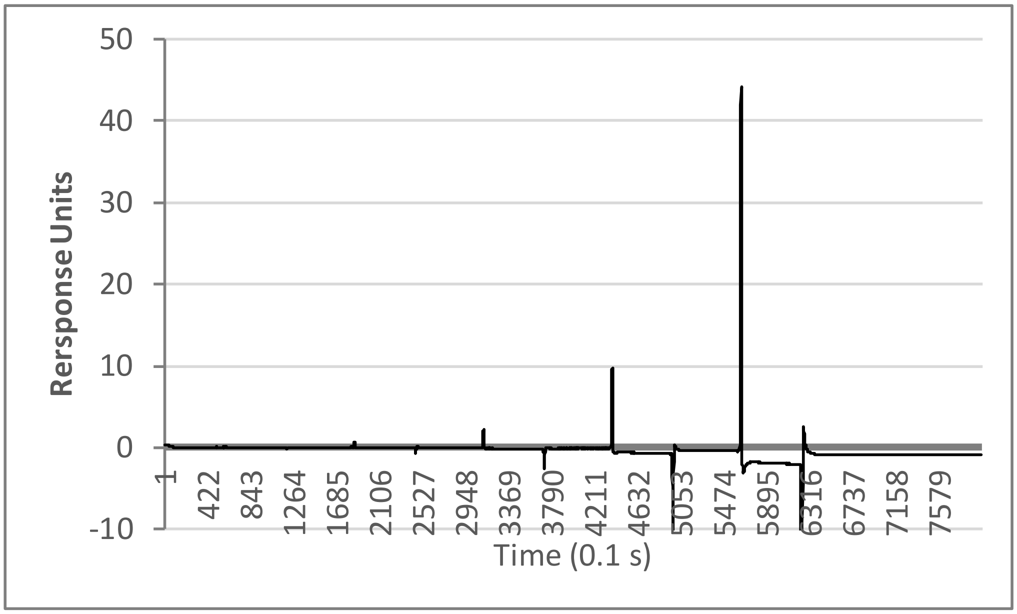

(M) REJO produced in HEK293T immortalized kidney cells interacting with the human rI-domain.

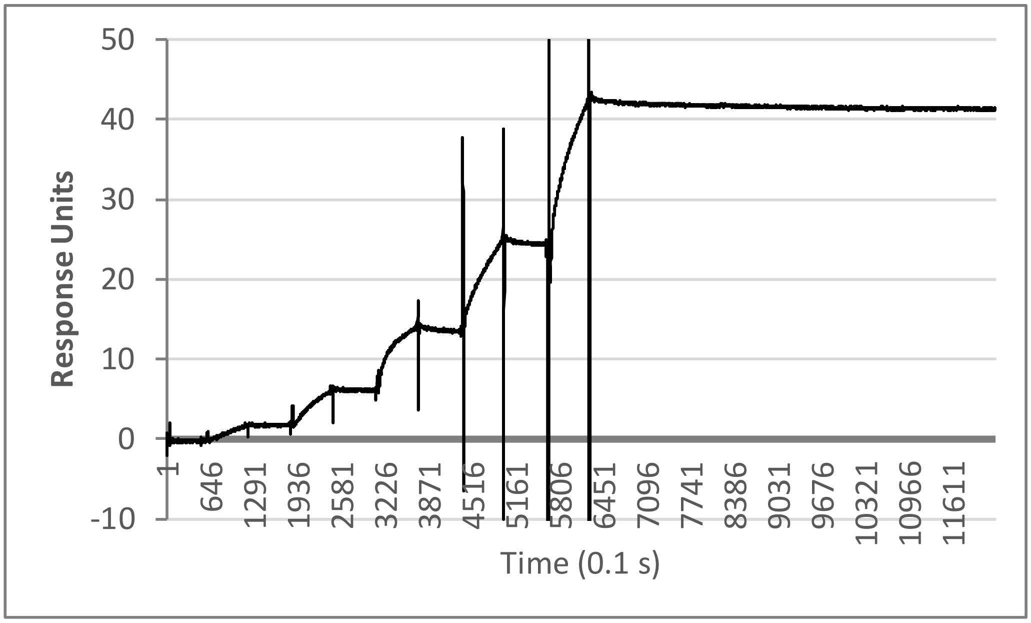

(N) PNGase F-treated REJO produced in HEK293T immortalized kidney cells interacting with the human rI-domain.

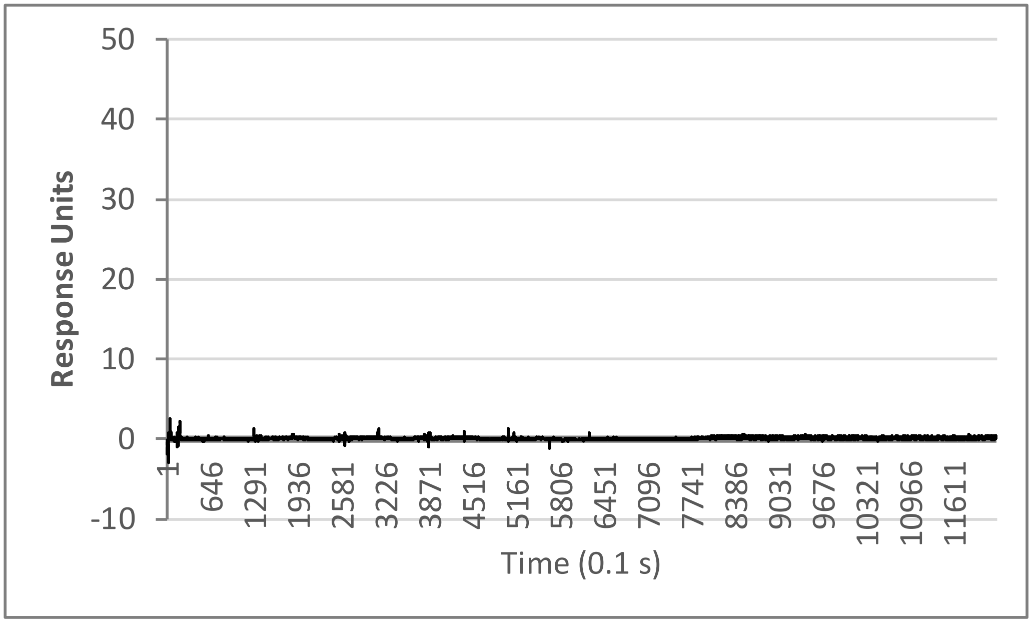

(O) REJO produced in HEK293T immortalized kidney cells interacting with the human rCR3.

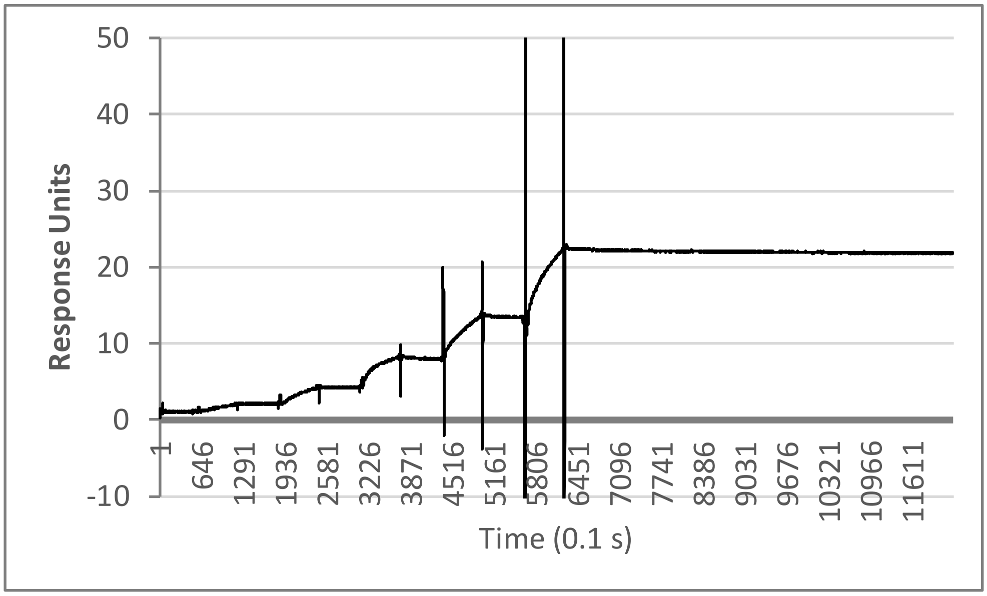

(P) PNGase F-treated REJO produced in HEK293T immortalized kidney cells interacting with the human rCR3.

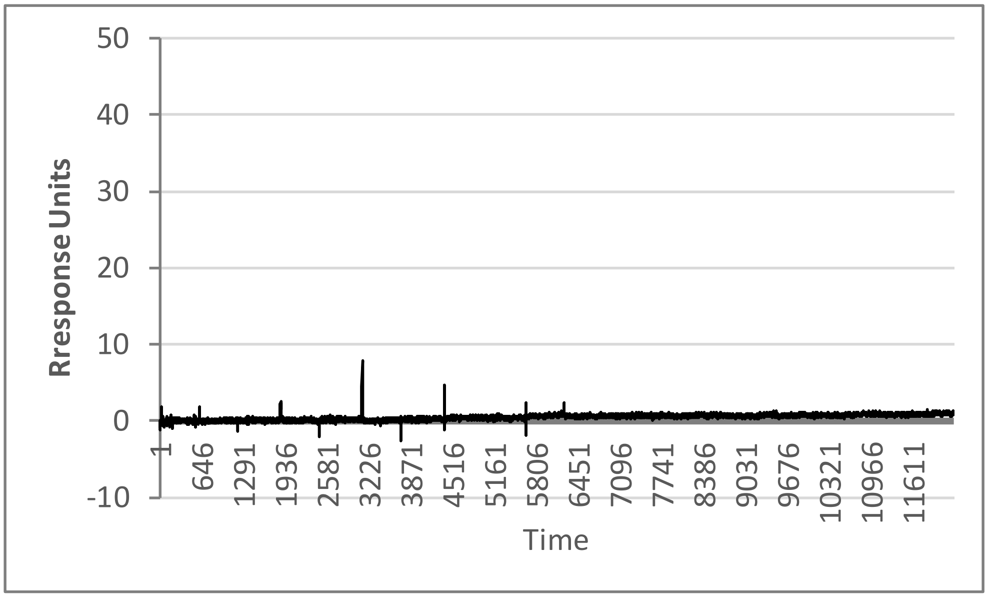

(Q) WITO produced in HEK293T immortalized kidney cells interacting with the human rI-domain.

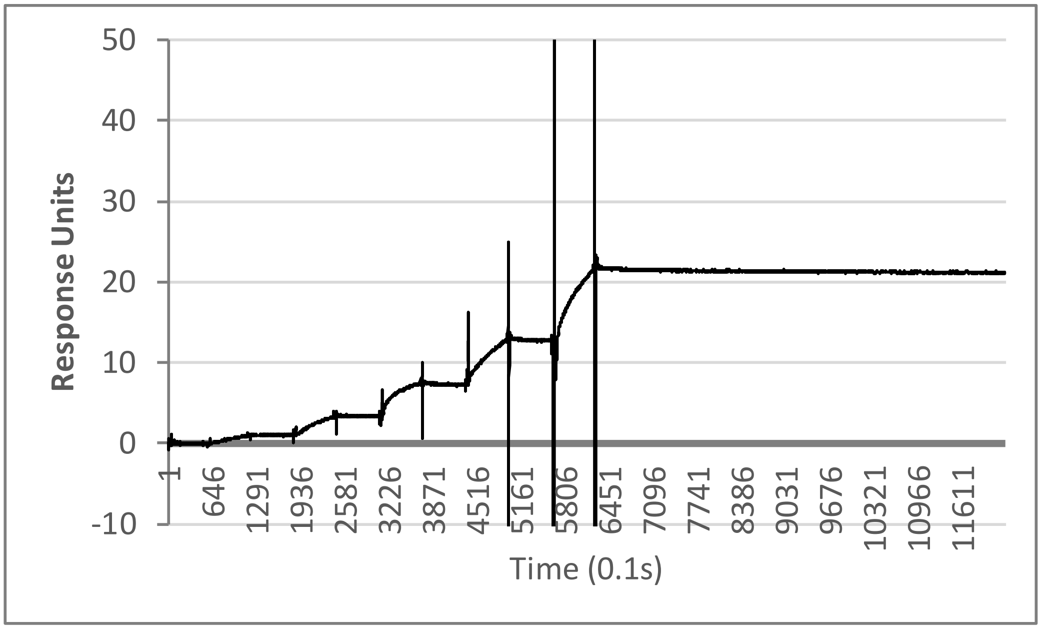

(R) PNGase F-treated WITO produced in HEK293T immortalized kidney cells interacting with the human rI-domain.

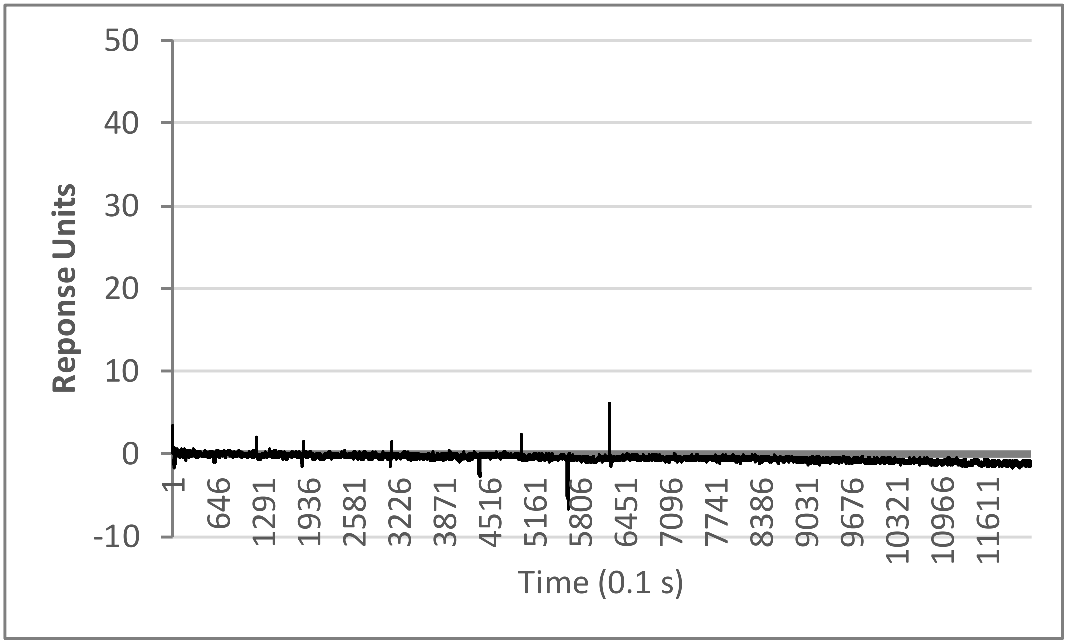

(S) WITO produced in HEK293T immortalized kidney cells interacting with the human rCR3.

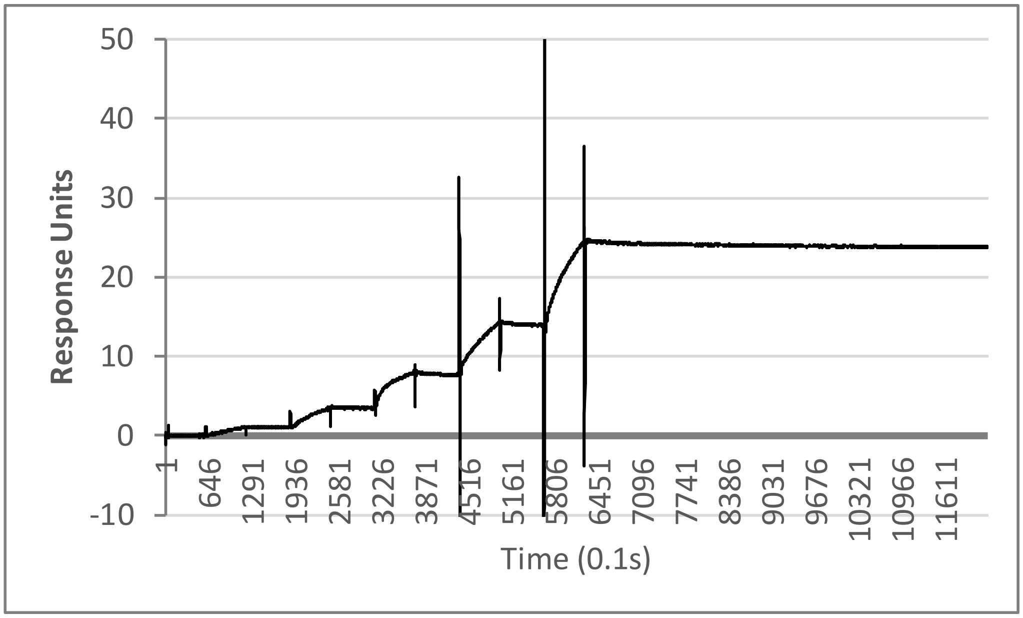

(T) PNGase F-treated WITO produced in HEK293T immortalized kidney cells interacting with the human rCR3.

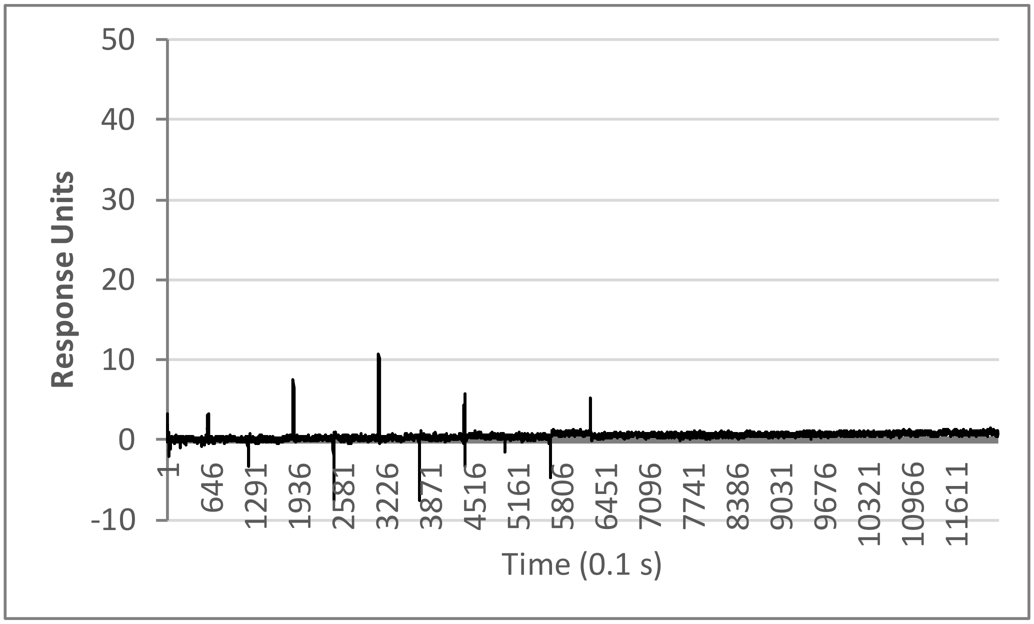

(U) WITO produced in peripheral blood mononuclear cells interacting with the human rI-domain.

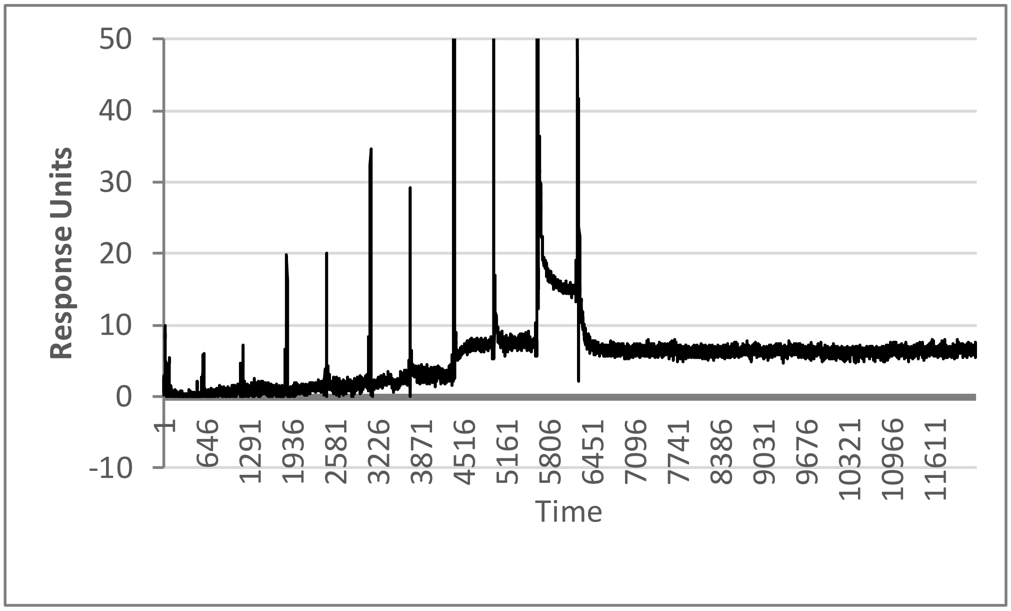

(V) PNGase F-treated WITO produced in peripheral blood mononuclear cells interacting with the human rI-domain.

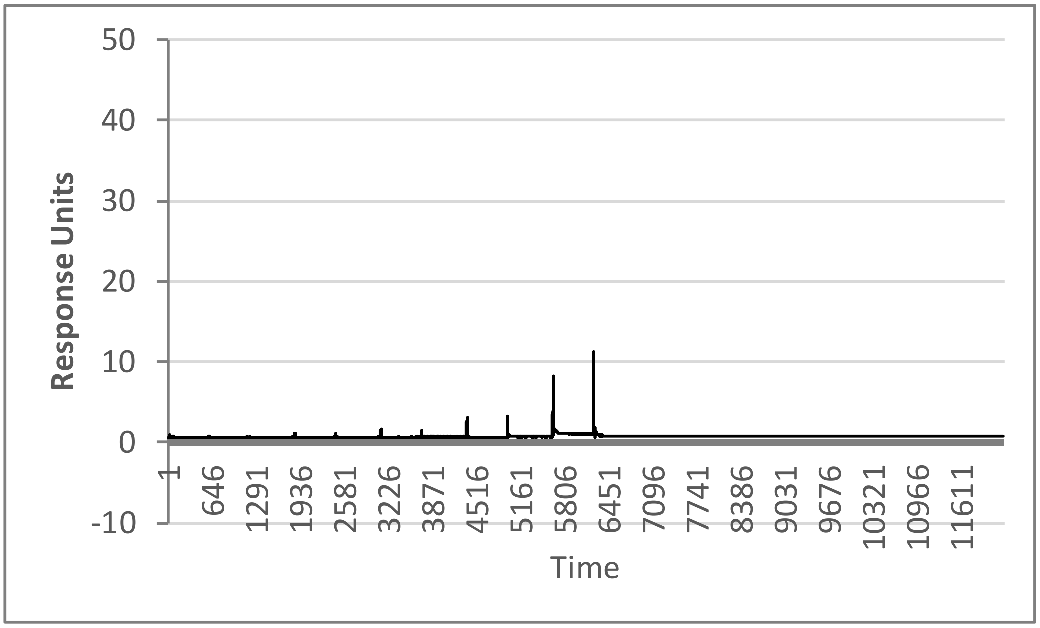

(W) WITO produced in peripheral blood mononuclear cells interacting with the human rCR3.

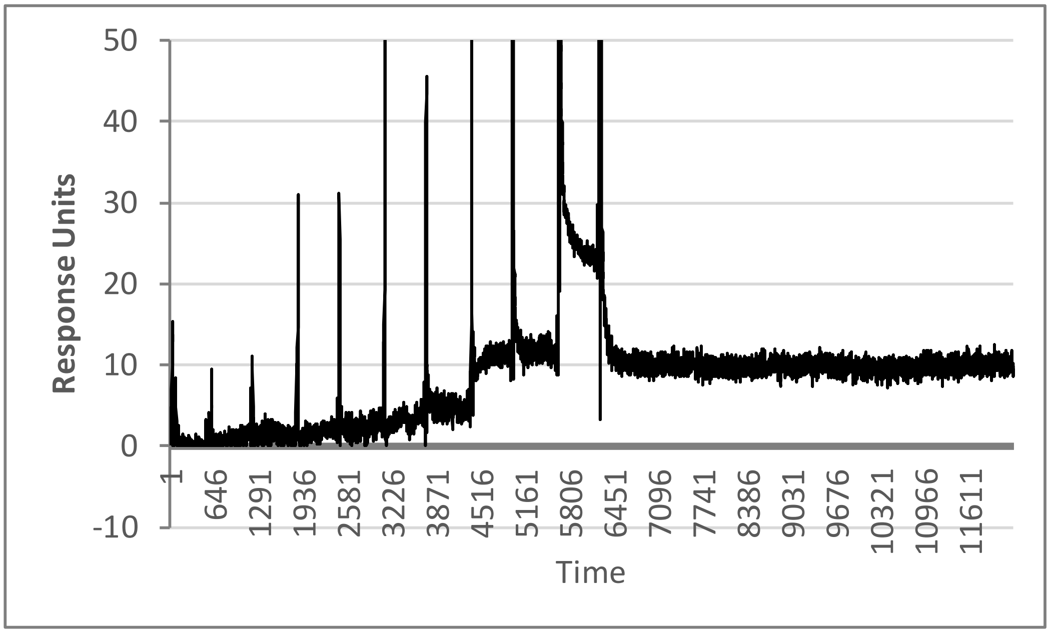

(X) PNGase F-treated WITO produced in peripheral blood mononuclear cells interacting with the human rCR3.

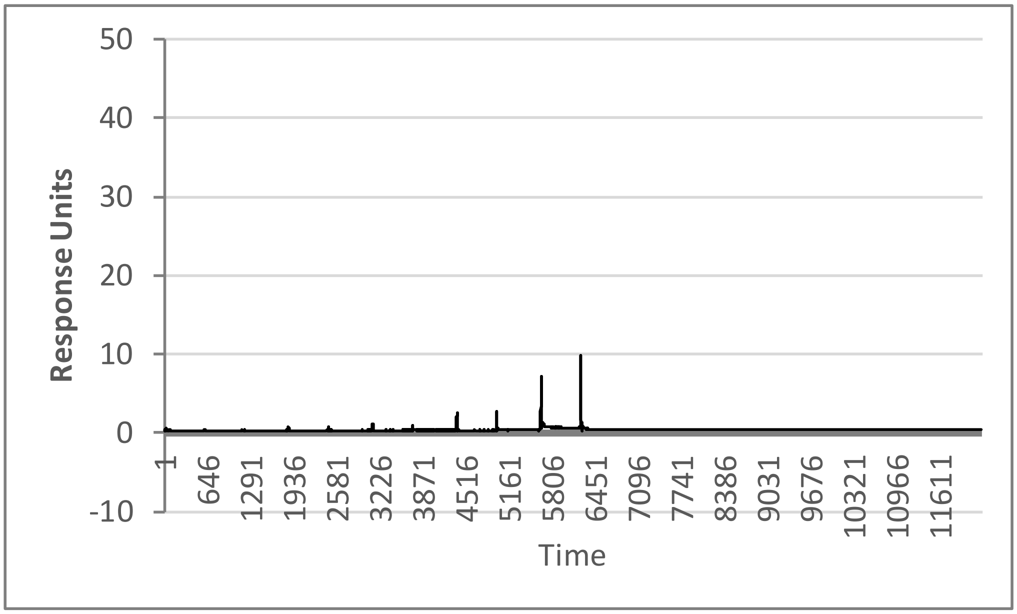

(Y) Man5 interacting with the rI-domain.

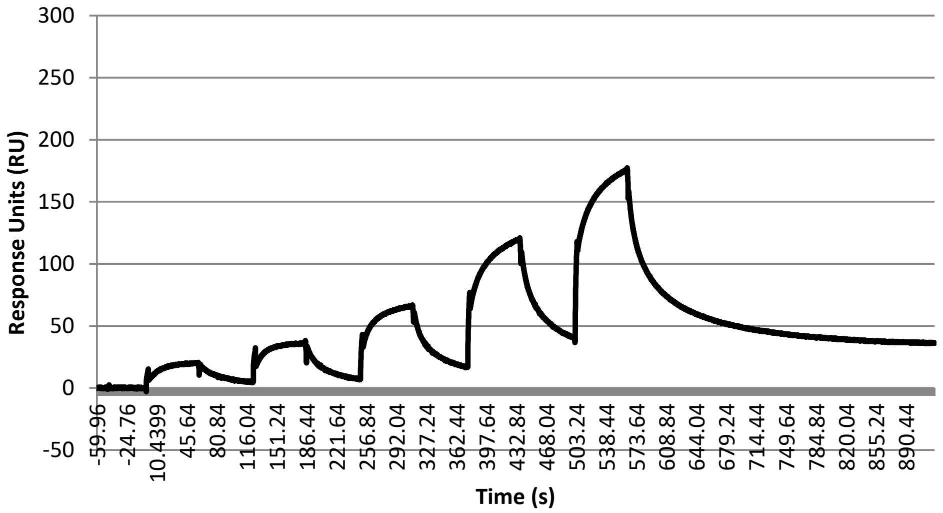

(Z) Man5 interacting with rCR3.

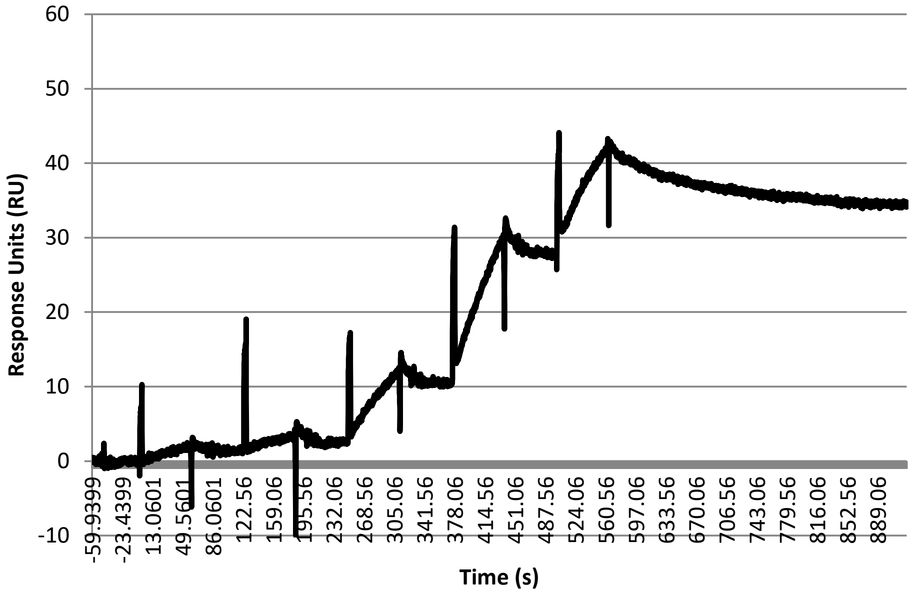

Figure S1. Representative sensorgrams of Table 1 interactions. *(A)* NL4-3 AD8 produced in HEK293T immortalized kidney cells interacting with the human rI-domain. *(B)* PNGase F-treated NL4-3 AD8 produced in HEK293T immortalized kidney cells interacting with the human rI-domain. *(C)* NL4-3 AD8 produced in HEK293T immortalized kidney cells interacting with the human rCR3. *(D)* PNGase F-treated NL4-3 AD8 produced in HEK293T immortalized kidney cells interacting with the human rCR3. *(E)* NL4-3 produced in HEK293T immortalized kidney cells interacting with the human rI-domain. *(F)* PNGase F-treated NL4-3 produced in HEK293T immortalized kidney cells interacting with the human rI-domain. *(G)* NL4-3 produced in HEK293T immortalized kidney cells interacting with the human rCR3. *(H)* PNGase F-treated NL4-3 produced in HEK293T immortalized kidney cells interacting with the human rCR3. *(I)* RPHA produced in HEK293T immortalized kidney cells interacting with the human rI-domain. *(J)* PNGase F-treated RPHA produced in HEK293T immortalized kidney cells interacting with the human rI-domain. *(K)* RPHA produced in HEK293T immortalized kidney cells interacting with the human rCR3. *(L)* PNGase F-treated RPHA produced in HEK293T immortalized kidney cells interacting with the human rCR3. *(M)* REJO produced in HEK293T immortalized kidney cells interacting with the human rI-domain. *(N)* PNGase F-treated REJO produced in HEK293T immortalized kidney cells interacting with the human rI-domain. *(O)* REJO produced in HEK293T immortalized kidney cells interacting with the human rCR3. *(P)* PNGase F-treated REJO produced in HEK293T immortalized kidney cells interacting with the human rCR3. *(Q)* WITO produced in HEK293T immortalized kidney cells interacting with the human rI-domain. *(R)* PNGase F-treated WITO produced in HEK293T immortalized kidney cells interacting with the human rI-domain. *(S)* WITO produced in HEK293T immortalized kidney cells interacting with the human rCR3. *(T)* PNGase F-treated WITO produced in HEK293T immortalized kidney

cells interacting with the human rCR3. *(U)* WITO produced in peripheral blood mononuclear cells interacting with the human rI-domain. *(V)* PNGase F-treated WITO produced in peripheral blood mononuclear cells interacting with the human rI-domain. *(W)* WITO produced in peripheral blood mononuclear cells interacting with the human rCR3. *(X)* PNGase F-treated WITO produced in peripheral blood mononuclear cells interacting with the human rCR3. *(Y)* Man5 interacting with the rI-domain. *(Z)* Man5 interacting with rCR3.
