# Supplementary material for: Complement Receptor 3 Mediates HIV-1 Transcytosis across an Intact Cervical Epithelial Cell Barrier: New Insight into HIV Transmission in Women
Source: mBio. 2022 Jan 11;13(1):e02177-21. doi: 10.1128/mbio.02177-21 (PMC8749410; doi:10.1128/mbio.02177-21)
Supplement: FIG S2 [file mbio.02177-21-sf002.pdf]

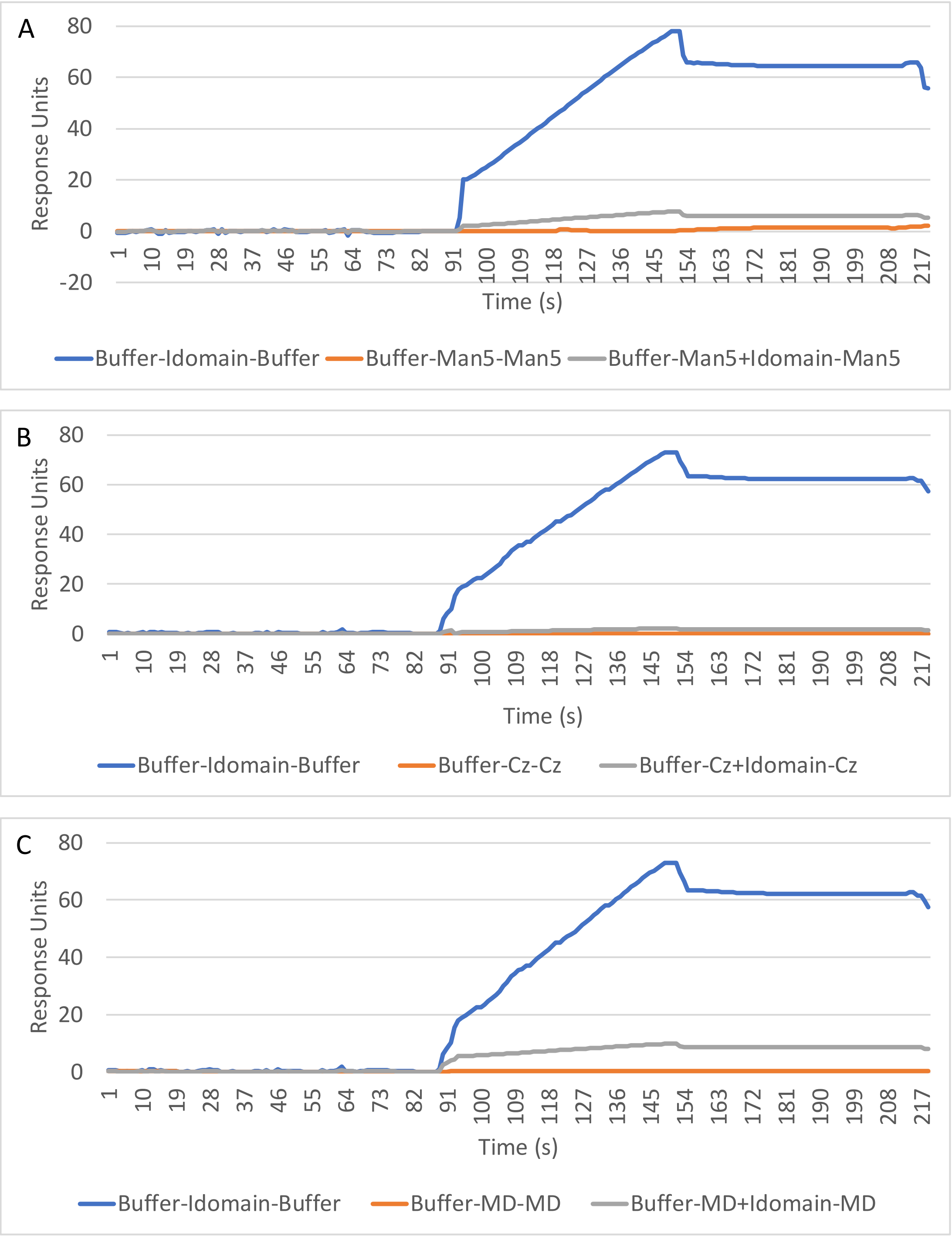
A
B
C
Response Units
Time (s)
80
60
40
20
0
-20
1
10
19
28
37
46
55
64
73
82
91
100
109
118
127
136
145
154
163
172
181
190
199
208
217
Buffer-Idomain-Buffer
Buffer-Man5-Man5
Buffer-Man5+Idomain-Man5
Buffer-Cz-Cz
Buffer-Cz+Idomain-Cz
Buffer-MD-MD
Buffer-MD+Idomain-MD

Figure S2. Representative competition curves in support of Figure 1. Competition between 1μM Man5 *(A)*, carbamazepine *(B)*, or α-methyldopa *(C)* and recombinant human CR3 I-domain at 1 μM concentration with immobilized HIV-1 WITO generated in primary human PBMCs.
