# Supplementary material for: Complement Receptor 3 Mediates HIV-1 Transcytosis across an Intact Cervical Epithelial Cell Barrier: New Insight into HIV Transmission in Women
Source: mBio. 2022 Jan 11;13(1):e02177-21. doi: 10.1128/mbio.02177-21 (PMC8749410; doi:10.1128/mbio.02177-21)
Supplement: FIG S3 [file mbio.02177-21-sf003.pdf]

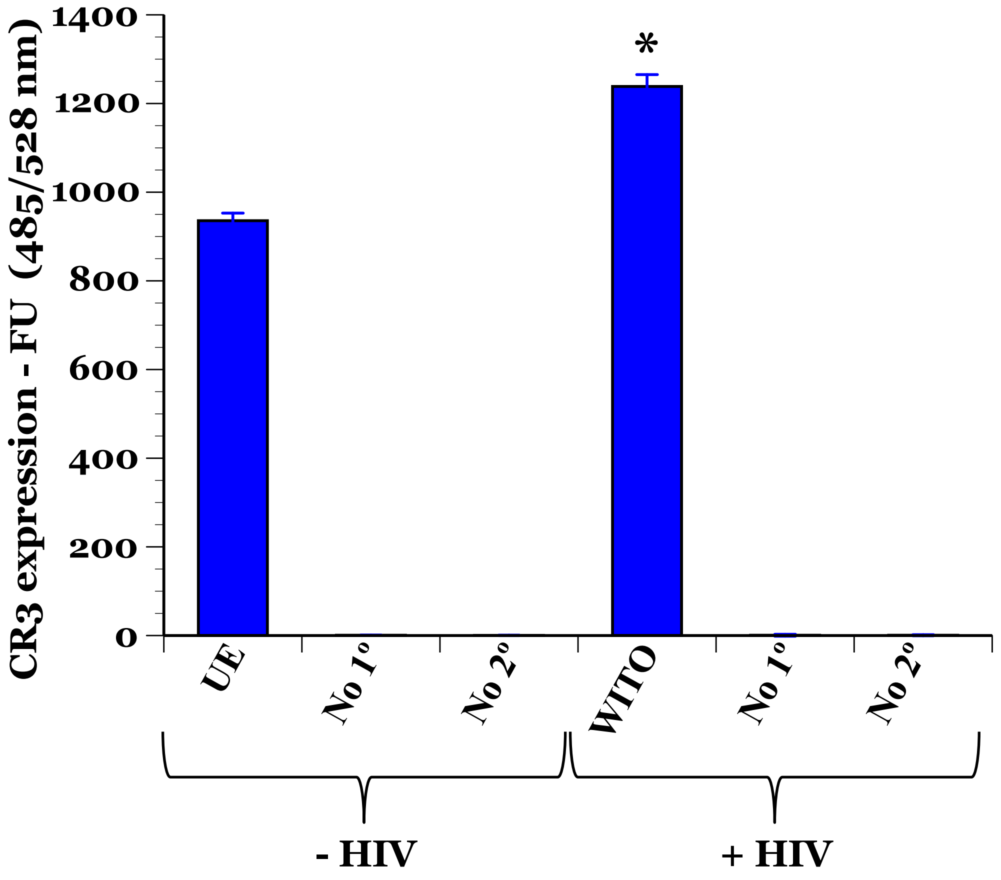

Figure S3. **HIV-1 exposure results in the increased expression of CR3 on the Pex cell surface.** Pex cells were exposed to HIV-1 strain WITO for 2h after which relative CR3 expression on the Pex cell surface was determined by immunolabeling CR3 and recording fluorescence units (FU). Pex cells not exposed to HIV (- HIV) expressed CR3, which was significantly increased following HIV exposure (+ HIV). Data shown represent the mean and variance of 3 assays performed in triplicate. UE - cells not exposed to HIV-1 strain WITO; No 1º - the primary antibody was omitted from the immunolabeling procedure; No 2º - the secondary antibody was omitted from the immunolabeling procedure; * - $p \leq 0.0001$ versus unexposed (-HIV) cells
