# Supplementary material for: Complement Receptor 3 Mediates HIV-1 Transcytosis across an Intact Cervical Epithelial Cell Barrier: New Insight into HIV Transmission in Women
Source: mBio. 2022 Jan 11;13(1):e02177-21. doi: 10.1128/mbio.02177-21 (PMC8749410; doi:10.1128/mbio.02177-21)
Supplement: FIG S4 [file mbio.02177-21-sf004.pdf]

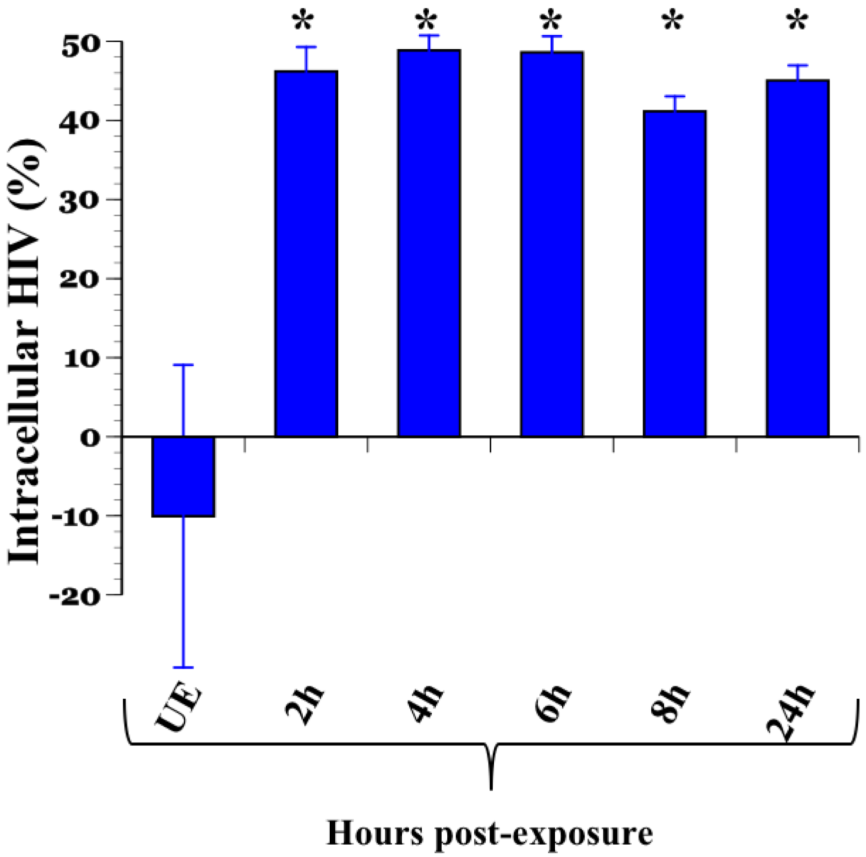

Figure S4. **HIV-1 can be internalized by Pex cells.** The relative amount of intracellular HIV-1 was measured over 24h following Pex cell exposure by using a double fluorometric immunolabeling technique to distinguish extracellular HIV from intracellular HIV, as described in the text. The percentage of intracellular HIV was then calculated and is shown on the y-axis. Background levels of fluorescence were recorded for Pex cells not exposed to HIV (UE). HIV-exposure resulted in HIV being found intracellularly, the level of which remained fairly stable over the time course assayed. Data shown represent the mean and variance of 3 assays performed in duplicate. * - $p \leq 0.0001$ versus Pex cells not exposed to HIV.
