# Supplementary material for: Complement Receptor 3 Mediates HIV-1 Transcytosis across an Intact Cervical Epithelial Cell Barrier: New Insight into HIV Transmission in Women
Source: mBio. 2022 Jan 11;13(1):e02177-21. doi: 10.1128/mbio.02177-21 (PMC8749410; doi:10.1128/mbio.02177-21)
Supplement: FIG S5 [file mbio.02177-21-sf005.pdf]

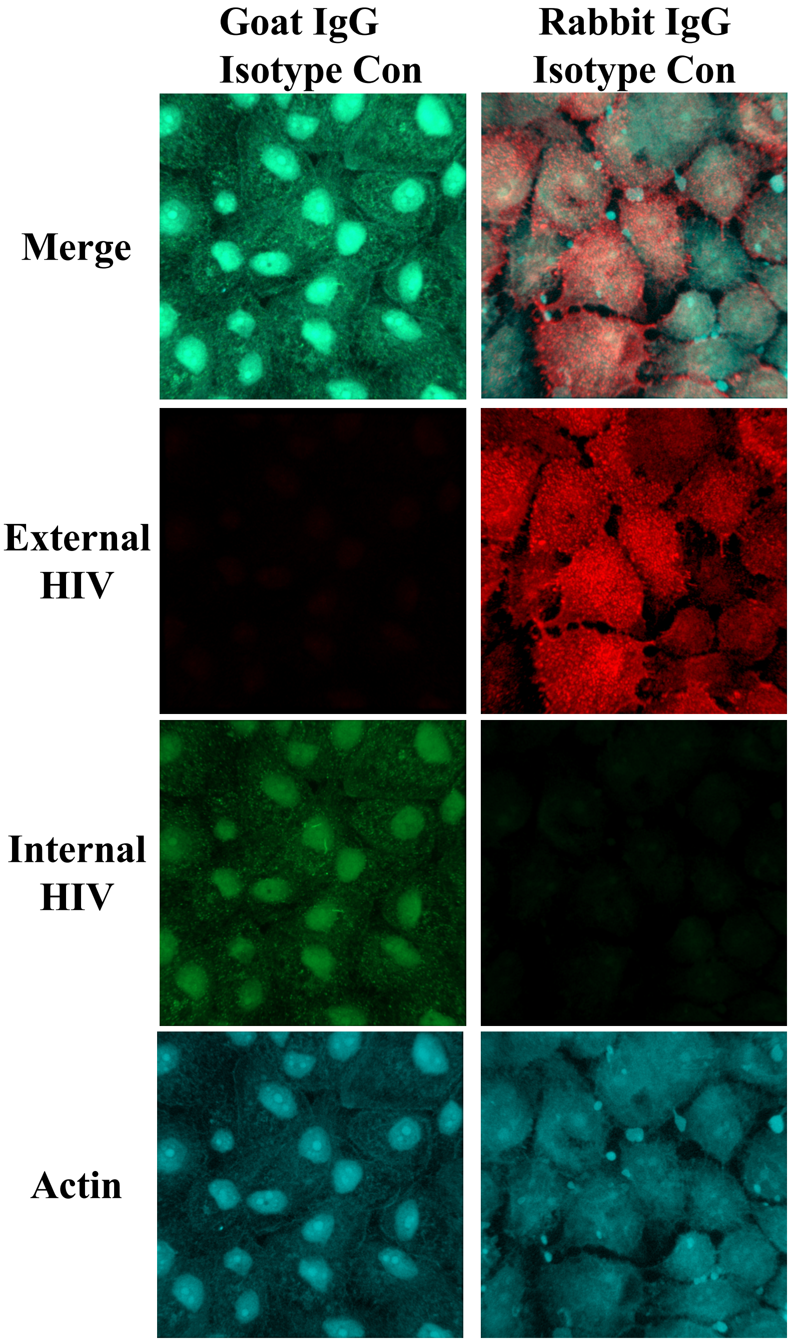

Figure S5. Assay controls for data shown in figure 6. Pex cells were incubated for 4h with HIV-1 strain WITO before double immunolabel processing for confocal microscopy, as described in the text. Right panel) External HIV-1 was immunolabeled using the goat polyclonal antibody, LS-C103187, which recognizes HIV p17, p24, p53, p64, p120, and p160, and an AF647-conjugated secondary antibody. Following permeabilization of host Pex cells, a rabbit IgG isotype control antibody, and an AF488-conjugated secondary antibody, were used to immunolabel internal/total virus. Internal virus was not recognized by the rabbit IgG control antibody, as indicated by the lack of a signal in the green channel. Left panel) Pex cells were incubated with a goat IgG isotype control antibody, and an AF647-conjugated secondary antibody. Pex cells were then permeabilized before incubation with the rabbit polyclonal antibody, LS-C486994, which recognizes HIV p24, and an AF488-conjugated secondary antibody. External virus were not recognized by the goat IgG control antibody, as indicated by the lack of a signal in the red channel. Data shown were obtained in parallel with data shown in figure 6, and demonstrate the specificity of the antibodies used to immunolabel both external and internal virus. For all coverslips examined, Alexa Fluor Plus 405 Phalloidin was used to stain host Pex cells. Magnification – 63X with oil emersion.
